# Mycelial communities associated with *Ostrya carpinifolia*, *Quercus pubescens* and *Pinus nigra* in a patchy Sub-Mediterranean Karst woodland

**DOI:** 10.1007/s00572-025-01220-9

**Published:** 2025-07-25

**Authors:** Tanja Mrak, Philip Alan Brailey-Crane, Nataša Šibanc, Tijana Martinović, Jožica Gričar, Hojka Kraigher

**Affiliations:** 1 Department of Forest Genetics and Physiology, Slovenian Forestry Institute, Večna pot 2, 1000 Ljubljana, Slovenia; 2 Department of Genetics, University of Georgia, C424 Life Sciences Building, Athens, GA 30602 USA

**Keywords:** Ectomycorrhizal fungi, Mesh bags, Exploration strategies, Hop-hornbeam, Pubescent oak, European black pine

## Abstract

Common mycorrhizal networks of ectomycorrhizal (EcM) fungi could be of great benefit to trees growing in the shallow soils of Sub-Mediterranean Karst ecosystems, potentially playing a crucial role in the survival of trees in this harsh environment. The first step to confirm the existence of such networks is to assess the extent and nature of symbiont sharing in the mycelial community. To address this question, we incubated in-growth mesh bags under the native *Ostrya carpinifolia* and *Quercus* *pubescens*, and the non-native *Pinus nigra*, over two consecutive years. In *Q. pubescens* and *P. nigra*, but not in *O. carpinifolia*, mycelium production was significantly higher in the year with higher spring precipitation, indicating the influence of climatic conditions, but also the identity of the host tree. We observed a complex interaction between tree species and sampling year in structuring the composition and diversity of mycelial communities. Local environmental conditions contributed additionally and were responsible for 21.46% of the community variation between samples. Although ~ 70% of fungal operational taxonomic units were shared across the studied tree species, distinct community compositions emerged, emphasizing the role of host tree specificity. *Q. pubescens* exhibited greater stability in EcM richness between sampling years, whereas *P. nigra* showed lower EcM richness, likely due to limited availability of compatible fungi and reliance on introduced fungal partners. Additionally, differences in EcM fungal exploration strategies were observed. *O. carpinifolia* and *Q. pubescens* mainly hosted non-specific EcM fungi with short distance exploration types. In contrast, EcM fungi of *P. nigra* had higher spatial spread, and were predominantly conifer specific. Overall, our results emphasize the importance of host specificity, soil parameters, spatial proximity, and climatic variability for the structuring of mycelial communities in fragmented forests.

**Supplementary Information:**

The online version contains supplementary material available at 10.1007/s00572-025-01220-9.

## Introduction

Soil fungi are involved in a wide range of ecosystem processes, such as the decomposition of organic matter, soil carbon (C) storage, and phosphorous (P) and nitrogen (N) cycling. On average, they represent between 55 to 89% of the total soil microbial biomass (Treseder and Lennon 2015). An important group of soil fungi in forest ecosystems are ectomycorrhizal (EcM) fungi, which form symbiotic associations with tree roots and obtain most of their carbon from their hosts, while delivering water and nutrients to plants (Lehto and Zwiazek 2011; Treseder and Lennon 2015; Bunn et al. 2024). The mycelium of EcM fungi contributes around 30% of soil microbial biomass (Högberg and Högberg 2002).

Mycelia of many EcM and saprotrophic fungi can spread vegetatively for considerable distances through soil (Klein and Paschke 2004; Cairney 2005), thereby taking up resources at the specific location and translocating them to other regions (Klein and Paschke 2004). Generally, most dominant EcM fungi occur in patches with diameter less than three meters (Lilleskov et al. 2004), but recorders may stretch across more than one hundred of meters (Fiore-Donno and Martin 2001; Molinier et al. 2016). Genets of saprotrophic basidiomycetes can cover several square metres to many hectares (Boddy et al. 2009). Certain species of EcM fungi can link individual plants of the same species or even hosts of distant lineages through extramatrical mycelium (EMM) (Anderson and Cairney 2007; Pickles and Simard 2017). These mycelial linkages, called common mycorrhizal networks may facilitate water and nutrient redistribution in drought-stressed ecosystems and promote the establishment of tree seedlings in these environments (Pickles and Simard 2017). The tendency to share symbionts with other plants may enlarge the biotic component of the plant niche (Taudiere et al. 2015). A high degree of symbiont sharing may facilitate seedling establishment, especially in environments where summer droughts decidedly affect the survival of seedlings (Taudiere et al. 2015). Moreover, benefits of common mycorrhizal networks are not exclusively limited to environments with periodic aridity, improved height growth and shoot biomass in seedlings with access to common mycorrhizal networks was also reported from humid environments (Cortese and Horton 2024). Recently, concerns have been raised regarding the overstatements of benefits of common mycelial networks, arguing that their ecological significance remains scientifically unproven (Karst et al. 2023; Irwin 2024). Therefore, further evidence is needed to confirm or reject whether the common mycorrhizal networks are widespread in forests. One of the first steps in this direction is to investigate whether different coexisting tree species share common EcM fungi in their EMM community.

The indeterminate filamentous nature of EcM fungi is a serious obstacle for the investigation of mycelia in the heterogeneous soil environment (Cairney 2005). The tiny and fragile nature of mycelia prevents their direct quantification and identification since they cannot be separated from soil or mycelium of other soil fungi (Anderson and Cairney 2007). This challenge led to the development of the mesh bag method (Wallander et al. 2001). Mesh bags are made of a fine mesh with size openings tiny enough to allow only the ingrowth of fungal mycelium while excluding roots. They are filled with acid washed silica sand to remove C sources and limit the colonisation of mesh bags only to EcM fungi (Wallander et al. 2001). However, recent studies have brought this assumption into question (Hagenbo et al. 2018; Fernandez 2021). Despite their drawbacks, mesh bags were successfully used in several C cycling studies (e.g. Hagenbo et al. 2017, 2018). On the other hand, next generation sequencing (NGS) approaches, such as metabarcoding, provide insight into the fungal community composition, but cannot always differentiate between active mycelium and dormant fungal structures, such as spores and sclerotia (Janowski and Leski 2023). Additionally, metabarcoding, a widely used NGS approach for fungal community studies, is semi-quantitative (Baldrian et al. 2013, Nguyen et al. 2015). Combination of both methods is used to taxonomically identify the mycelium that develops inside mesh bags (Kjøller 2006). Communities of EcM fungi when considered from the perspective of their mycelial systems in soil may be quite different from those identified on the colonized root tips (Anderson and Cairney 2007).

Locally, the composition of microbial communities in topsoil is determined by the spatial heterogeneity in terms of soil and litter chemistry (organic matter content, pH, the content of N and other nutrients), vegetation composition and activity (Baldrian 2017). EcM community composition is strongly affected by the host identity (Bogar and Kennedy 2013). More than 50% of EcM species is specific at certain taxonomic level (Tedersoo et al. 2024). These drivers are accompanied by stochastic effects on the assembly of the microbial community (Baldrian 2017). One of them is a priority effect (Kennedy et al. 2009), the timing and order of species arrival at the site, with coexistence, deterministic exclusion and history-dependent exclusion as possible outcomes (Song et al. 2021). The strength of priority effect may depend on the mode of colonization, with lower possibility to establish from spores due to restrained energy source contained in spores compared to common mycorrhizal networks where mycelial growth may be fuelled by the plant hosts (Johnson 2015).The community structure is also shaped by the dispersal ability of fungal spores which is limited by distance in EcM fungi (Lilleskov and Bruns 2005; Peay et al. 2012).

Different EcM fungi produce different amounts of EMM (Anderson and Cairney 2007). Exploration types of ectomycorrhizae were introduced to describe the extent of EMM produced in the form of emanating hyphae and rhizomorphs (i.e. aggregations of parallel-oriented hyphae) to increase the volume of exploited soil and colonize new roots (Agerer 2001). Contact exploration type is characterized by a smooth mantle and only a few emanating hyphae, while short-distance exploration type has numerous emanating hyphae that do not extend far from the root tip. The medium-distance exploration type features undifferentiated or slightly differentiated rhizomorphs, with the amount of emanating hyphae depending on the sub-type (fringe, mat, and smooth). Finally, the long-distance exploration type forms rather smooth ectomycorrhizae with few but highly differentiated rhizomorphs (Agerer 2001). However, recent findings suggest that exploration types of EcM fungi may not be good predictors of soil foraging (Jörgensen et al. 2023).

Our study focused on harsh Sub-Mediterranean Karst environment in Slovenia, which is characterized by shallow soils and frequent summer droughts. Common mycorrhizal networks of EcM fungi could greatly benefit trees growing in this environment due to the role that common mycorrhizal networks have in the redistribution of water (Egerton-Warburton et al. 2008). The patchy nature of woodland in this area allows to study the parameters, which are structuring the mycelial community composition at the local level, such as host identity, geographical distance and potential spatial spread of ectomycorrhizal fungi present as mycelia, thereby providing basic knowledge to support the further studies on the belowground connectivity in this ecosystem. The prevailing EcM hosts present in the Sub-Mediterranean Karst in Slovenia are pubescent oak, (*Quercus pubescens Willd.*), hop-hornbeam (*Ostrya carpinifolia* Scop.) and black pine (*Pinus nigra* Arnold). The non-native *P. nigra* was the only species for which the afforestation efforts in the past were successful in this area (Kranjc 2009) but is becoming less and less desired due to its high susceptibility to fire, different abiotic disturbances and insect infestations (Diaci et al. 2019).

A patchy secondary woodland ecosystem, which we selected for our study appears as separate groups of trees intermixed with meadows or pastures. Three before-mentioned tree species, which dominate separate groups of woody vegetation in the area, were investigated for their EMM communities in mesh bags for two subsequent years. Mesh bags were selected rather than bulk soil sequencing to differentiate the fungi present in mycelium forms from other fungi as mycelium can be easily separated from sand within mesh bags. To our knowledge, research on EcM communities of *O. carpinifolia* is very limited and focused mainly on *Tuber* cultivation (see Benucci et al. 2011; Baciarelli Falini et al. 2012; Moser et al. 2017). We hypothesized that the core EcM community in EMM would remain relatively stable between years but shifts in community composition may occur in response to interannual climatic variability. We hypothesized that tree species in the patchy woodland would share some EcM fungi in their EMM communities, with a higher degree of sharing expected between phylogenetically closer species (e.g. *O. carpinifolia* and *Q. pubescens*, both belonging to Fagales) compared to *P. nigra*. Finally, we expected that *P. nigra* would rely more on EcM fungi with higher spatial spread, given the growth habit of its root system, as its thicker fine roots may not penetrate tiny soil pores and therefore depend more heavily on EcM hyphae for nutrient and water uptake.

## Materials and methods

### Location and focal study species

The study was performed at Podgorski Kras (45.541714ºN, 13.916392ºE, 430 m a. s. l.), a karst region in SW Slovenia which is characterized by a Sub-Mediterranean climate. The maximum precipitation occurs in the fall and late spring, whereas the low precipitation periods occur in the transition from the winter to spring and in July/August (Ogrin 1996). The heterogeneous patchy woodland (meadows and pastures intermixed with groups of trees) present there originates from the abandonment of pastures in the area after WWII (Zorn et al. 2015). Spontaneous natural afforestation was dominated by the native tree species such as *Quercus pubescens*, *Fraxinus ornus* L. and *Ostrya carpinifolia*, along with the introduced *Pinus nigra*, which spread from nearby plantations established through past human intervention. Notably, *O. carpinifolia* primarily colonizes sites where dry stonewalls were historically constructed to clear rocks from agricultural land and protect it from wind erosion, particularly around sinkholes or between separate pasture plots. These sites are characterized by very shallow soils. Nine plots, three per tree species of interest, were selected (Figure S1). Each plot measured approximately 200 m^2^. Coordinates of the plots are given in Table S1.

### Mesh bag preparation and exposure

To assess the EMM development in-situ, the in-growth mesh bag method was used (Wallander et al. 2001). The triangular mesh bags with sides of the triangle measuring *a* = *b* = 10 cm, and *c* = 14.5 cm were prepared from inox 304L woven metal cloth (Jutotissu Kft., Budapest, Hungary) with 50 µm wire diameter and pore openings of 46 µm diameter to allow only fungal hyphae ingrowth excluding plant roots (Figure S2a). Mesh bags were filled with 25 g of silicate sand of granulation 0.71–1.25 mm (Aquasil Filtersand, Quarzwerke, Melk, Austria) to achieve approximately 1 cm thickness of the mesh bag. The sand was first autoclaved, then washed with 2.5 M HCl to remove organic matter and subsequently with ultrapure water to remove any traces of added acid. The removal of organic matter was performed to eliminate a potential pre-existing nutrient source in the sand. Ingrowth mesh bags were inserted into the soil of the nine plots (three per tree species) in two series over the two subsequent years, first series from 28 March 2019 till 15 April 2020 (385 days) and the second from 17 June 2020 till 15 July 2021 (394 days). The exposure period of approximately one year was selected according to Hagenbo et al. (2018), suggesting that longer incubation times (around one year) counteract the initial disturbance effect and better reflect the EcM fungal community assembly in soil.

At each plot, we selected nine individuals of the representative tree species where mesh bags were installed approximately 0.5 m away from the tree stem. At each of the nine trees per plot, three replicates of mesh bags were installed to ensure enough mycelium for analyses. In total, 243 mesh bags (3 plots × 3 species × 9 individual trees × 3 mesh bags) were installed in each campaign. They were inserted into the soil at approximately 10–15 cm depth by lifting intact bulk soil with a small spade and placing it back over the bags to minimize the disturbance of the soil layers. Collected mesh bags were kept frozen at −20 °C until further processing. At the time of processing, mesh bags were thawed, opened, and the quantity of mycelium visually estimated (Figure S2b) using a scale adapted from Wallander et al. (2001) by applying six classes: 0—no mycelia present; 1—sparse mycelia present; 2—small amount of mycelium present; 3—mycelia present all-over, but no aggregation of the sand particles; 4—plenty of mycelia present and some aggregation of the sand particles; and 5—plenty of mycelia present and sand particles aggregated to a large extent. After that, sand with mycelium was mixed with distilled water from which the mycelium floating on water was picked out with tweezers and transferred into an Eppendorf tube. Additionally, sand was checked under the binocular microscope to collect the remaining mycelium. Mycelium from the three replicated mesh bags was pooled. The collected mycelium was freeze-dried, and homogenized using MillMix20 mixer mill (Domel, Železniki, Slovenia).

### Meteorological conditions

Meteorological parameters for the observed period and 25-year period (1992–2017) were obtained from the Slovenian Environment Agency (meteo.arso.gov.si; accessed 19.1.2024). For precipitation, data were obtained from a nearby meteorological station Kozina (45.6042ºN, 13.9319ºE; 484 m a.s.l.), while air temperatures were extrapolated from other three closest meteorological stations (Portorož, Godnje and Postojna) as for Kozina no temperature measurements exist. Total cumulative precipitation amounted to 1484 mm and 1694 mm for the first and second campaign, respectively, while monthly precipitations in comparison to 25-year averages are presented in Figure S3. The 25-year average yearly precipitation for the area was 1299 mm and the average temperature was 11.8 °C. In the time of our study no summer drought typical for this area occurred.

### Soil analyses

Soil samples were collected in the close vicinity of the mesh bag installation points. Due to the stony terrain, samples were collected with a knife from the top 10–15 cm of the soil profile. For each plot nine soil subsamples were collected, which were pooled into three composite samples per plot (joining three times three subsamples) for physical–chemical analyses. The labels of mesh bags, from the vicinity of which soil samples were collected and pooled, were recorded. We measured pH, total, mineral and organic C (C_org_), total N, carbonates, extractable P, extractable K, Ca and Mg. The following standardized methods were used: SIST ISO 10390:2006: Soil quality—Determination of pH, SIST ISO 10694:1996: Soil quality—Determination of organic and total C after dry combustion (elementary analysis), SIST ISO 13878:1999: Soil quality—Determination of total nitrogen content by dry combustion ("elemental analysis"), SIST EN ISO 10693:2014: Soil quality—Determination of carbonate content—Volumetric method (ISO 10693:1995), SIST EN ISO 6878:2004: Water quality—Determination of phosphorus—Ammonium molybdate spectrometric method (ISO 6878:2004)—analogous for soil, SIST ISO 11466:1996: Soil quality—Extraction of trace elements soluble in aqua regia.

### Molecular methods

To quantify the total fungal community, DNA was extracted from 250 mg of homogenized lyophilized mycelium using DNeasy Power Soil Pro kit (Qiagen, Venlo, Netherlands), following the manufacturer’s instructions. Fungal communities were quantified using Illumina MiSeq NGS sequencing of ITS2 region amplicons. To produce amplicon libraries for Illumina MiSeq NGS, the ITS2 fragment was first amplified by PCR using Q5® Hot Start High-Fidelity 2X Master Mix (New England BioLabs, Ipswich, Massachusetts, USA) and the primer pair ITS7f (Ihrmark et al. 2012) and ITS4r (White et al. 1990). Forward and reverse primers were modified to contain Illumina specific overhang adapter sequences. PCR was carried out in a 25 µl reaction volume with 2 µl of DNA template, 12.5 µl of Master Mix and 1.25 µM of each primer. PCR conditions were 98 °C for 30 s followed by 25 cycles at 98 °C for 10 s, 57 °C for 20 s and 72 °C for 20 s and final elongation at 72 °C for 2 min on an Applied Biosystems Veriti Thermal Cycler (Thermo Fisher Scientific, Waltham, Massachusetts, USA). PCR products were purified using Agencourt AMPure XP magnetic beads (Beckman Coulter, Brea, California, USA). Illumina sequencing adapters and multiplex indexes were attached using the Nextera XT Index Kit (Illumina, San Diego, California, USA) following Illumina’s recommended protocols. Secondary PCR products were purified using Agencourt AMPure XP magnetic beads, before quantification using a Quant-iT PicoGreen dsDNA Assay Kit (Invitrogen). Equimolar concentrations (4 nM) of successfully amplified samples were pooled, and the pooled library was quality checked with Agilent High Sensitivity DNA kit. Sequencing was performed on the Illumina MiSeq (2 × 300 cycles, 15% PhiX) at Faculty of Medicine of University of Maribor.

### Bioinformatics

Primer sequences were trimmed from the raw sequence reads using cutadapt (Martin 2011), discarding any sequences with mismatches in the primer sequence (other than degenerate bases). Forward and reverse sequences were then quality filtered, trimmed, and pair-end aligned using fastp (Chen 2023). Briefly, sequences were discarded if more than 20% of bases had a quality score below Q30. A sliding-window approach was applied from the 5′ to 3′ direction, trimming bases when the average quality score within the window dropped below Q20. Only sequences longer than 40 nucleotides after trimming were retained for further analysis. Sequences passing the quality filtering and trimming steps were then pair-end aligned with a minimum detected overlap of 15 base pairs. We implemented an additional post-overlap length filter using Bash functions to retain sequences between 234 and 407 nucleotides in length (99.905% remained after length filtering based on the length distribution). We then concatenated sequences from all and dereplicated duplicate sequences with VSEARCH (Rognes et al. 2016). To remove flanking regions from the ITS2 gene region we used ITSx (Bengtsson-Palme et al. 2013), retaining only fungal ITS2 sequences. The remaining sequences were then denoised and clustered into OTUs at 97% similarity with VSEARCH. We used an OTU-based approach as there are multiple recognized issues with applying an amplicon sequence variant approach on ITS sequences (see Kauserud 2023). Centroid sequences were chimera checked, using the ‘uchime3’ algorithm implemented in VSEARCH, and sequences were then mapped to remaining centroids at 97% similarity to create an OTU table. Raw sequence data were deposited in SRA (BioProject PRJNA1243991).

Taxonomy was assigned to OTUs using a dual approach for maximum coverage. Taxonomy was first assigned using blast (Altschul et al. 1990) against the UNITE database (Abarenkov et al. 2024) release 10.0 2024–04-04 (all eukaryotes) containing only known genera with putative ecological assignment based on Põlme et al. (2021) assessed from SEED2 webpage (www.biomed.cas.cz/mbu/lbwrf/seed/). A second round of taxonomy assignment was performed with the CONSTAX2 (Liber et al. 2021) assignment method with the UNITE database. This approach derives a consensus taxonomy from BLAST, the RDP Naïve Bayesian classification algorithm, and the SINTAX classification algorithm. We carried out CONSTAX2 assignments with a confidence score of 0.7 for all three approaches, and an additional percentage identity cut-off of 0.9 for the BLAST approach. This was used to verify the initial BLAST assignments and manually curate the final dataset. OTUs were filtered from the dataset if they could not be assigned to the fungal kingdom through the ‘high level taxonomy’ assignment function of CONSTAX2. Where assignments were made to OTUs that were not captured through SEED2 BLAST annotation, the CONSTAX taxonomy was used, and functional annotations were updated based on prior ecological assignments of the genus where appropriate. Where a taxonomic rank was not able to be assigned at a given level, it was determined as ‘Unclassified’ for the purpose of taxa aggregation when appropriate. For analyses the data was split into three subsets covering the total fungal community, the EcM fungal community, and the saprotrophic fungal community.

### Data analysis

All analyses and plot generation were carried out in R (*v 4.3.1*) implemented through Rstudio (*v 2023.6.1*).

Soil parameters (Figure S4) were analysed using linear models considering Species as the fixed variable to determine differences between soils associated with each tree species.

Mycelium production was analysed using a cumulative link model (CLM) in *ordinal* package to examine the effects of Year and Tree species x Year. The model was based on 398 observations, with 88 excluded due to missing data.

OTUs were aggregated into three major ecological guilds as previously described (EcM fungi, plant pathogens, soil saprotrophs, per Põlme et al. (2021)) for statistical analysis of the total relative abundances of each guild across tree species and year through ANOVA. For the EcM guild, we further considered the relative abundances of species and genera assigned to different exploration types, covering contact, short-distance (SD), medium-distance (MD) and long-distance (LD) exploration types (Agerer 2001, Agerer 2006, Tedersoo and Smith 2013, Suz et al. 2014, Agerer and Rambold 2004–2025).

Observed OTU richness and Shannon’s index of diversity (H’) were calculated using *phyloseq* (*v1.44.0,* McMurdie and Holmes 2013)*.* Both variables were first tested for normality through a Shapiro-Wilkes test and analysed through ANOVA as they are robust to violations of normality particularly with large sample sizes. To determine whether rarefaction was necessary for downstream analyses we performed a correlation analysis between our observed OTU richness (*t* = 0.11, *p* = 0.91, r^2^ = 0.009) and H’ (*t* = −3.1, *p* = 0.002, r^2^ = 0.26) against sequencing depth. This was done for the total fungal community as the ectomycorrhizal subset is derivative of this and variation in the proportion of the community that is ectomycorrhizal would be confounded. While the correlation was significant between H’ and sequencing depth, the effect size of such was very weak and in fact negative rather than positive. Upon visual inspection this appears mostly driven by a biological effect stemming from 2021 samples having lower evenness in despite having higher sequencing depths than in 2020, rather than a technical issue associated with capture. We therefore believe it is prudent to maintain our data in a non-rarefied form per McMurdie and Holmes (2013).

To compare overall community dissimilarity, PERMANOVA analysis was performed with *vegan* using Bray–Curtis distances between samples. Overall community composition was also visualized through principal coordinate analysis (PCoA) of the same Bray–Curtis distances.

Statistical analyses associated with microbiome data were conducted using one of two linear models tested through ANOVA (*nlme)* or PERMANOVA (*vegan, v.2.6–4)* analysis. The first model tested the contribution of tree species and year of collection on observed OTU richness, H’ and Bray–Curtis distances: *[variable* ~ *tree species x year]*. As differences between tree species may be either driven or even masked by within-species variation based on field location, a second model was constructed to more finely consider the plot that samples were collected from irrespective of species identity: *[variable* ~ *plot x year].* Where significant main or interaction effects were seen (p < 0.05), post-hoc pairwise comparisons were carried out using the *emmeans* (ANOVA post-hoc) and *pairwise adonis* (*v.0.4.1,* PERMANOVA post-hoc) packages, with false discovery rate/Benjamini–Hochberg corrections for multiple testing.

We complemented our PCoA analyses with additional transformation-based redundancy analyses (tb-RDA) to determine how underlying soil properties and geographic distance between plots contributed to the community composition. Geographic distances were incorporated into the tb-RDA through the calculation of principal coordinates of neighbour matrices (PCNM) vectors. We further tested spatial variability between samples through Mantel correlations of Bray–Curtis distances and Euclidean spatial distances.

Indicator species analysis was implemented by the *indicspecies* package (*v.1.7.14*) at both the OTU and genus level. The number of shared and unique taxa were also visualized using upset plots (*MicrobiotaProcess* package *v.1.12.4*).

## Results

### Mycelium quantification

**Fig. 1:**
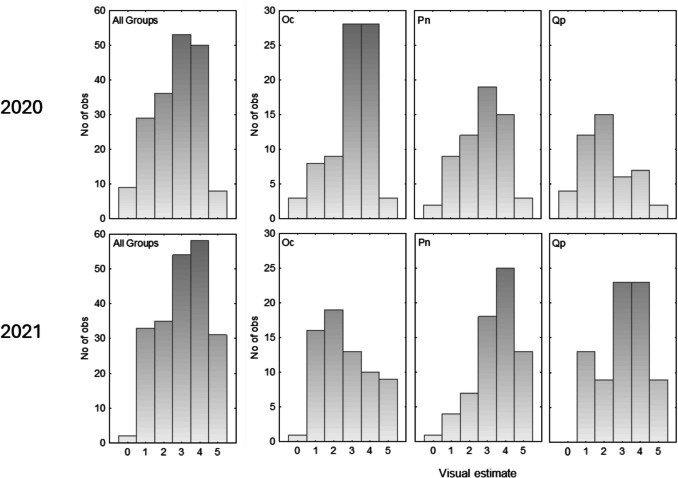
Frequencies of visual estimates of EMM quantity for recovered mesh bags from the two campaigns (2020 and 2021), for all tree species together (all groups) and each tree species separately (*Oc*, *O. carpinifolia*; *Pn*, *P. nigra*; *Qp*, *Q. pubescens*)

From the total number of mesh bags installed in the soil the recovery (Fig. 1) for the first campaign was 76.1%. The lowest recovery was for *Q. pubescens*, where 43.2% of bags were missing due to wild boar browsing, followed by 25.9% missing bags for *P. nigra* and 2.5% for *O. carpinifolia*. In the second campaign, the recovery of mesh bags improved to 87.7% of the total. At that time, 4.9% mesh bags were missing for *Q. pubescens*, and 16.0% for both *P. nigra* and *O. carpinifolia*. Considering all mesh bags, the most frequent visual estimations of mycelium quantity were 3 and 4 in both campaigns. The percentage of mesh bags with visual estimate 5 was higher in the second campaign (14.6% of all recovered mesh bags) compared to the first campaign (4.3% of all recovered mesh bags). Indeed, the mycelium quantity over all three species was significantly higher in 2021 (Estimate = + 2.591, *p* = 0.0010). However, the year 2021 was associated with a significant decrease in mycelium production relative to 2020 for *O. carpinifolia* (Estimate = − 0.637, *p* = 0.033). On the other hand, both *P. nigra* (Estimate = + 1.711, p < 0.001) and *Q. pubescens* (Estimate = + 1.994, *p* < 0.001) showed significant positive interactions with year 2021 suggesting increased mycelium production in 2021 relative to 2020.

### Study-wide statistics of OTUs

After quality filtering and denoising 4,568,529 sequences were generated across 142 samples. Two samples with low sequencing depth (< 1000 sequences) were removed from the analysis. The remaining samples contained a minimum of 5,998 and maximum of 104,489 sequences (median 26,920). A total of 3425 OTUs with 97% cut-off were generated, of which 901 could be assigned a species level (36.89% of dataset total sequences), and 1987 could be assigned a genus level (76.63% of dataset total sequences). Of those that could not be assigned a species or genus, an additional 414 could be assigned a family-level identification (8.8% of dataset total sequences).

### Functional groups of the mycelia-forming fungi colonizing mesh bags

**Fig. 2:**
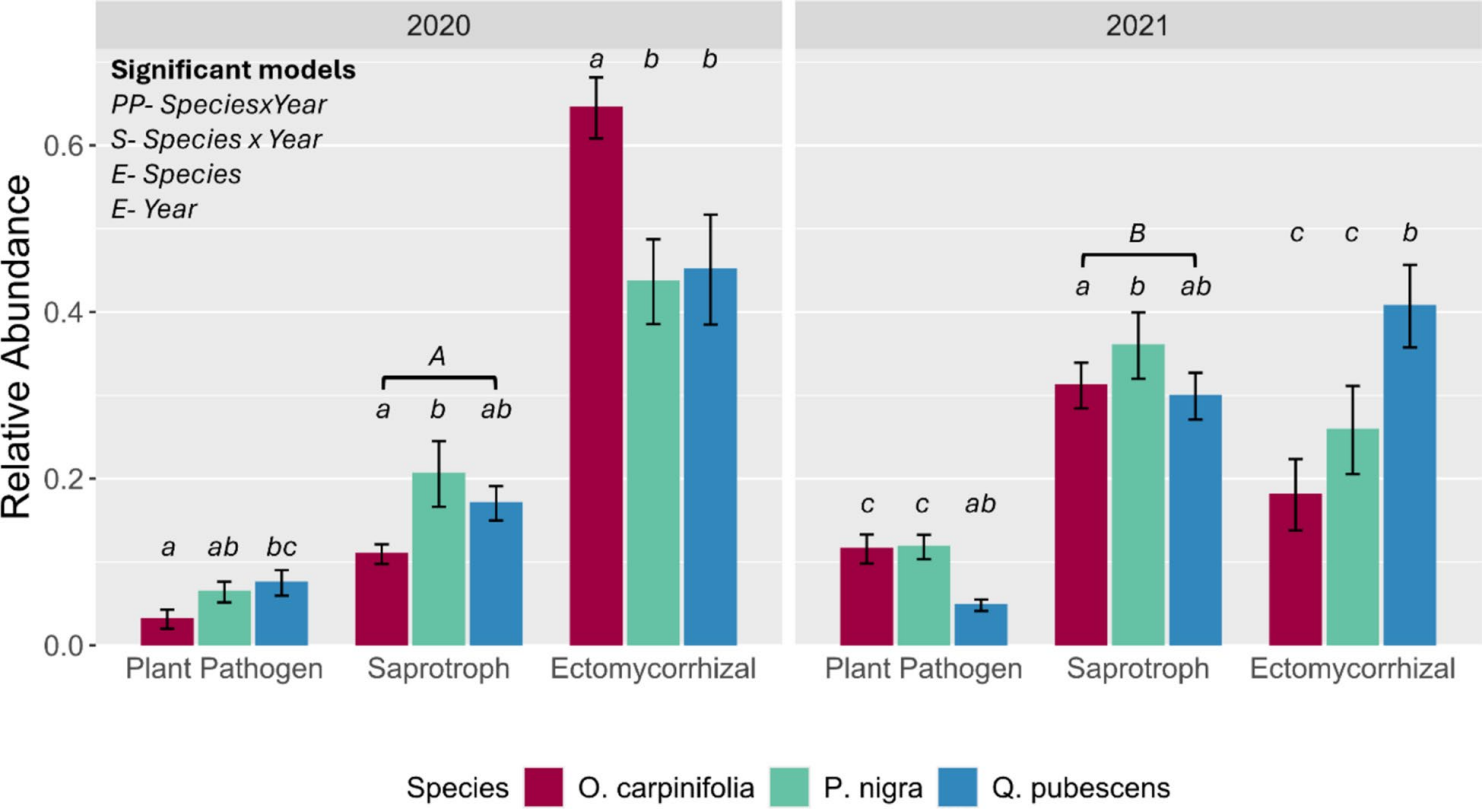
Mean relative abundances of fungal functional groups inside mesh bags incubated beneath *Ostrya carpinifolia* (n(2020) = 26, n(2021) = 23), *Pinus nigra* (n(2020) = 23, n(2021) = 24) and *Quercus pubescens* (n(2020) = 19, n(2021) = 26) in 2020 and 2021. Number of replications (*n*) per year is given in brackets. Letters represent groupings of statistical similarity after *p*-value correction (fdr). For the saprotroph functional group lowercase letters represent between species groups, and uppercase letters denote that relative abundances are different between years regardless of plant species identity. For ectomycorrhizal and plant pathogen functional groups, letters are derived from species x year interactions

There were 290 OTUs (representing 44.06% of sequences) classified as ‘EcM’ and 1277 OTUs (representing 21.06% of sequences) classified as exclusively ‘saprotrophic’. For 1215 OTUs (representing 23.5% of sequences) a putative function could not be assigned. The remaining 643 OTUs (23.5% of sequences) were assigned other putative functions including mixed saprobe-classifications, plant pathogens and other mycorrhizae (Fig. 2). Linear modelling revealed that the relative proportions of all OTUs belonging to the EcM, plant pathogen, and saprotroph guilds were highly variable between sampling years and across the associated tree species (Fig. 2, Table S4). The relative abundances of plant pathogens and EcM fungi were driven by an interaction between species and year. The relative abundance of plant pathogens increased between 2020 and 2021 for both *O. carpinifolia* and *P. nigra* but remained statistically consistent in *Q. pubescens* between years. The opposite relationship was seen in EcM fungi, where abundances decreased between 2020 and 2021 for *O. carpinifolia* and *P. nigra*, but again remained consistent between years in *Q. pubescens.* In 2020, EcM fungi were more abundant in *O. carpinifolia* than both other tree species. In 2021 however, EcM fungi were more abundant in *Q. pubescens* than both other tree species which was driven by between-year fluctuations in abundance for these two species against the relatively consistent abundances seen in *Q. pubescens.* For saprotrophic fungi, relative abundances were variable across tree species but were not informed by the interaction of the two variables. Saprotroph abundance was generally increased in 2021 relative to 2020, and post-hoc testing confirmed that saprotroph relative abundance was significantly higher in mycelial bags associated with *P. nigra* than *O. carpinifolia*, while *Q. pubescens* had intermediate abundances regardless of year.

### Exploration types of EcM fungi colonizing mesh bags

**Fig. 3:**
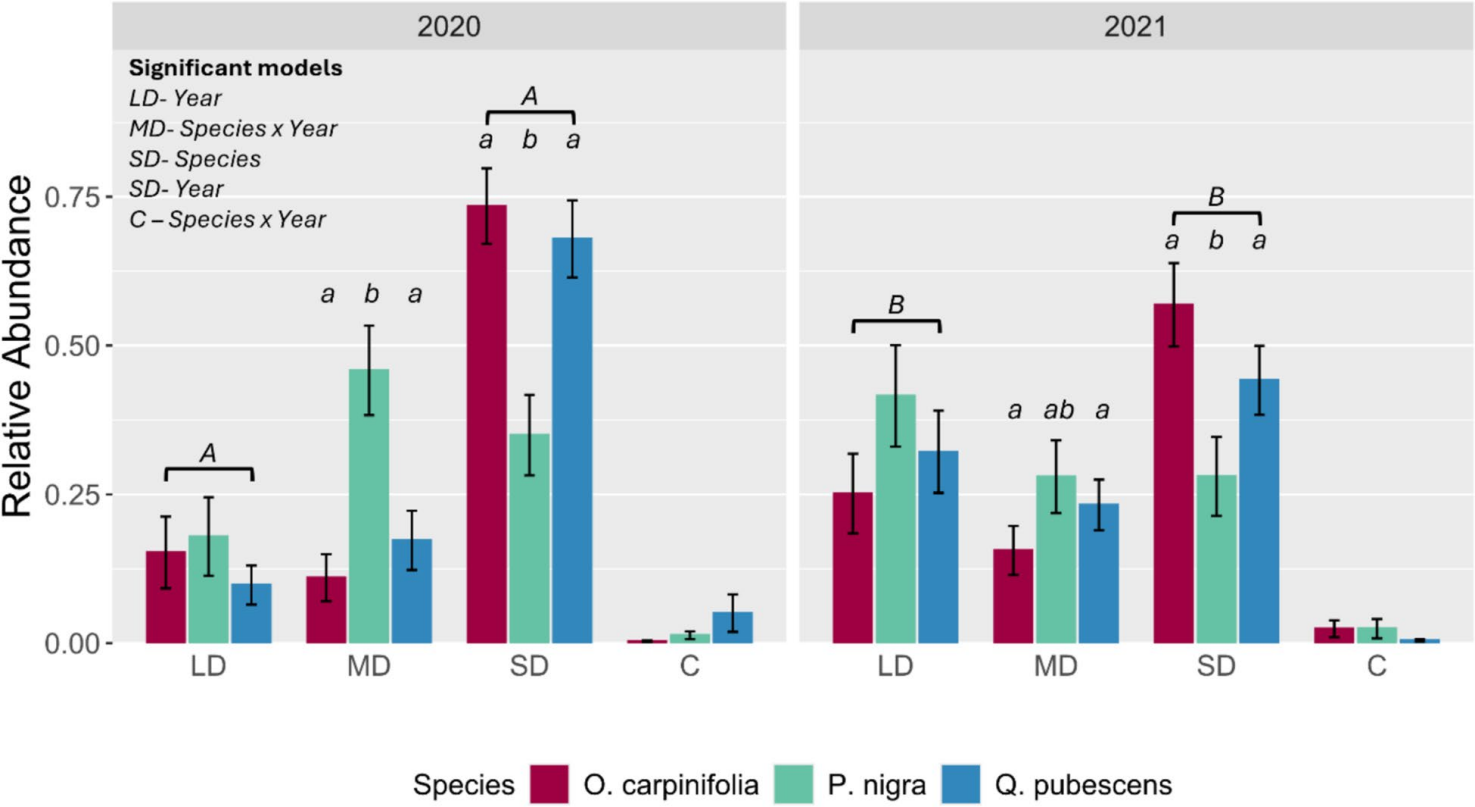
Mean relative abundances of exploration types of EcM fungi detected in mycelial communities of mesh bags incubated beneath *Ostrya carpinifolia* (n(2020) = 26, n(2021) = 23), *Pinus nigra* (n(2020) = 23, n(2021) = 24) and *Quercus pubescens* (n(2020) = 19, n(2021) = 26) in 2020 and 2021. Exploration types are long-distance (LD), medium-distance (MD), short-distance (SD) and contact (C). Number of replications (n) per year is given in brackets. Letters represent groupings of statistical similarity after p-value correction (fdr). For the LD and SD exploration types, lowercase letters represent between species groups where appropriate, and uppercase letters denote that relative abundances are different between years regardless of plant species identity. For MD exploration type, letters are derived from species x year interactions. There are no letter groupings associated with C as there was no statistical support for such in post-hoc testing

Across the whole experiment ectomycorrhiza associated with SD exploration were the most abundant (51.06 ± 2.97%), followed by LD (23.84 ± 2.81%), MD (23.27 ± 2.31%), and contact exploration types (1.82 ± 0.57%) in descending order (Fig. 3). The relative abundance of ectomycorrhiza belonging to the SD exploration type was different between tree species (Table S5), being generally less abundant in mesh bags associated with *P. nigra* (31.60% ± 4.71), than *O. carpinifolia* (65.80 ± 4.80%) and *Q. pubescens* (54.20 ± 4.62%). Across all three species, fungi with SD exploration decreased in relative abundance between 2020 and 2021 (Table S5). Fungi associated with the LD exploration type alternately increased in relative abundance between 2020 and 2021 (Table S5) but did not exhibit any species-specific associations (Table S5). The relative abundances of MD and contact exploration type fungi were driven by interactions between associated tree species and year (Table S5). Contact-associated fungi displayed small increases in relative abundance between 2020 and 2021 in mesh bags associated with *P. nigra* and *O. carpinifolia* and decreased in mesh bags associated with *Q. pubescens*. These associations however were not found to be statistically significant in pairwise comparisons after p-value correction, suggesting that while there may be a pattern it is too subtle and variable within species to make firm conclusions from. Fungi associated with MD exploration type were in greater relative abundance in mesh bags associated with *P. nigra* (2020: 45.80 ± 7.50, 2021: 28.0 ± 6.12) relative to those associated with *O. carpinifolia* (2020:11.0 ± 3.95; 2021:15.6 ± 4.12) and *Q. pubescens* (2020:17.3 ± 4.99,2021:23.2 ± 4.27) only in 2020. For relative abundances of subtypes of MD fungi see Figure S7.

### Alpha diversity

The interaction effect of tree species and sampling year were significant determinants of observed OTU richness for total fungi and saprotrophic fungi, while the two parameters individually were associated with variation in the OTU richness of EcM fungi with no significant interaction (Table S6). The Shannon diversity of the total fungal community was significantly affected by the interaction between tree species and year and for saprotrophic fungi was significantly affected only by tree species. No significant variation in Shannon diversity was found for EcM fungi across tree species or year (Table S6).

**Fig. 4:**
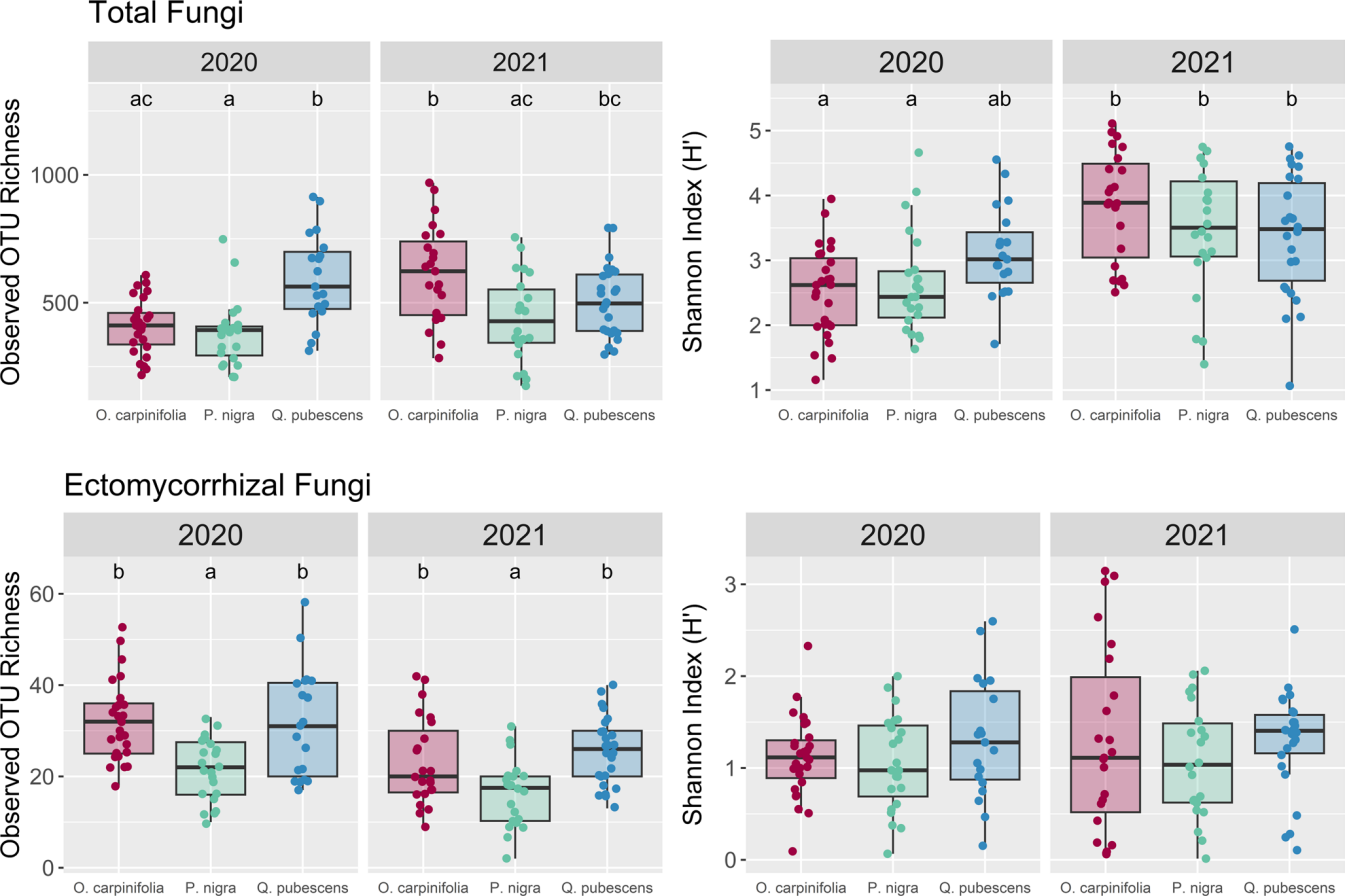
Boxplots displaying the distribution of observed OTU richness and Shannon diversity index (H’) for total fungi and the EcM subset. Boxes cover the 25th-75th percentile of each group’s distribution, with the median represented as a thick bar within the boxes. Extending lines from the box denote the 1.5 interquartile range of the 25th and 75th percentile. Significantly different values (*p* < 0.05) are marked with different letters. Note the different scale for each subset. Replications per subset were as follows: *O. carpinifolia* (n(2020)= 26, n(2021)=23), *P. nigra* (n(2020)=23, n(2021)=24) and *Q. pubescens* (n(2020)= 19, n(2021)=26)

In 2020 *O. carpinifolia* (mean richness = 408.85 ± 20.76; mean Shannon index = 2.51 ± 0.13) and *P. nigra* (mean richness = 379.65 ± 26.61; mean Shannon index = 2.63 ± 0.16) had significantly lower observed OTU richness and Shannon diversity index for total fungi than *Q. pubescens* (mean richness = 594.53 ± 40.31; mean Shannon index = 3.12 ± 0.16, Fig. 4, Table S6). However, in 2021 both alpha diversity metrics of *O. carpinifolia* (mean richness = 613.39 ± 39.04; mean Shannon index = 3.85 ± 0.18) and *P. nigra* (mean richness = 437.05 ± 35.92; mean Shannon index = 3.47 ± 0.21) increased and evened out with *Q. pubescens* (mean richness = 506.88 ± 27.19; mean Shannon index = 3.39 ± 0.18). For the EcM subset, the OTU richness was the lowest for *P. nigra* (mean richness *P. nigra* = 19.04 ± 1.10, *O. carpinifolia* = 27.98 ± 1.42, *Q. pubescens* = 28.11 ± 1.48, Fig. 4, Table S5), while no significant differences were observed in diversity. Recorded richness for the EcM subset was generally lower in 2021 (mean richness = 28.48 ± 1.23) compared to 2020 (mean richness = 21.92 ± 1.06, Table S6). Saprotroph subset followed the pattern for total fungi in terms of OTU richness, while diversity remained constant among both campaigns (Figure S5, Table S6).

### Beta diversity and community composition

**Fig. 5:**
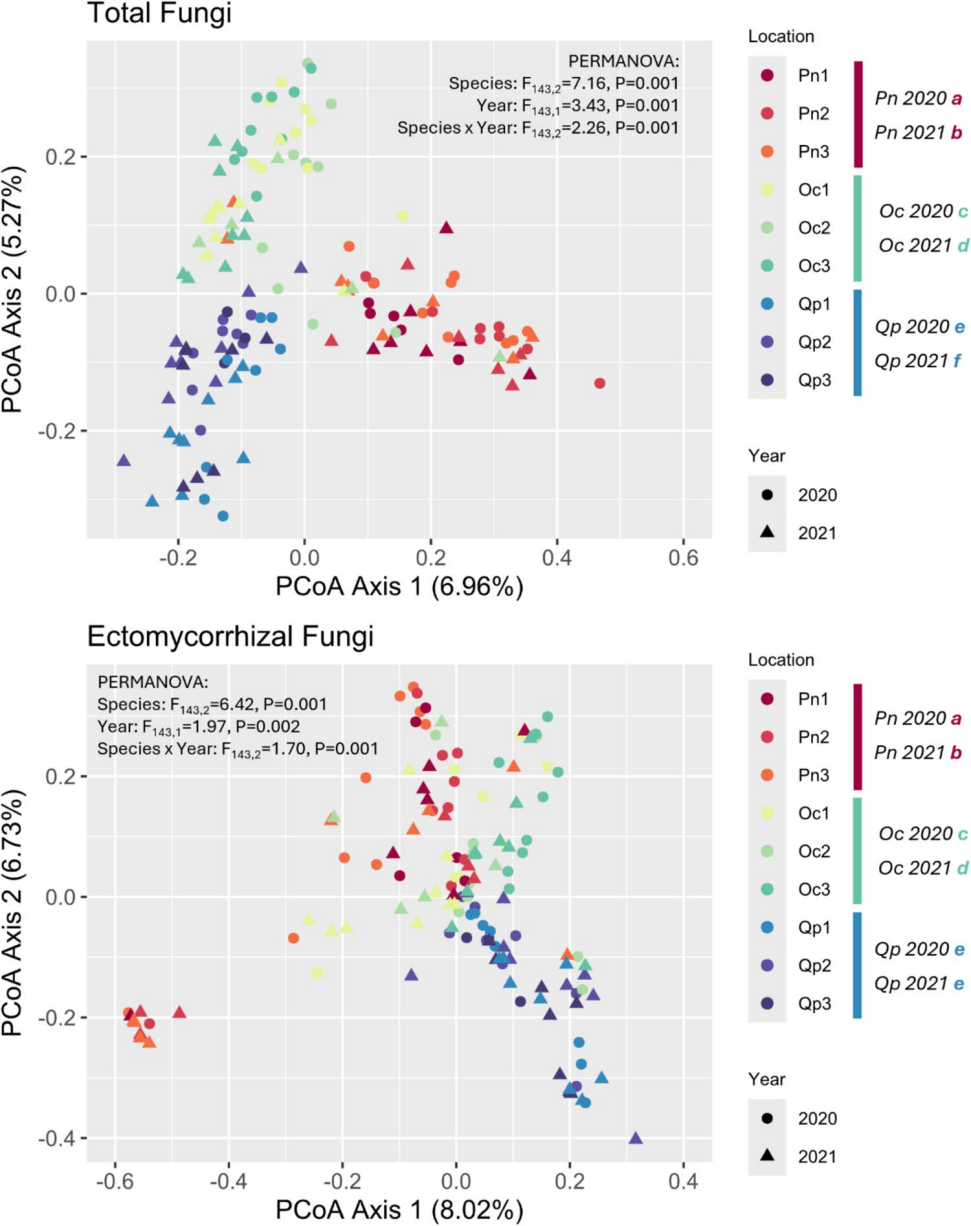
PCoA of total and EcM fungal communities in mesh bags. Communities from different tree species and plots are marked with different colours (for locations of plots see Figure S1), while communities from different sampling campaigns are shown by different shapes. Letters next to Species x Year combinations represent groupings of statistical similarity after posthoc pairwise PERMANOVA analysis with fdr corrections

The composition of both the total fungal community and EcM subset were found to be structured by the interaction between tree species and sampling year which explained 28.93% and 22.36% of between-sample variation respectively (Fig. 5). For both datasets, tree species contained consistently distinct community assemblies. For the total fungal community, all species and year combinations were distinct from one another whereas when considering the EcM subset, the community composition of fungi associated with *Q. pubescens* was consistent across both sampling years while those associated with *O. carpinifolia* and *P. nigra* were not (Fig. 5). There was also a significant degree of intra-specific variation in community assembly when considering sampling location (Table S7), though this explained only 7.99–9.91% of intra-species between-sample variation depending on tree species and dataset. The relative high importance of between-species variation over intra-species variation is demonstrated through Fig. 5 where the ordination reveals three well established groups that corresponded to tree species for the total fungal community rather than within-species plot location. For the EcM community, *Q. pubescens* associated fungi formed a clearly separate group from those associated with other tree species, while *P. nigra* and *O. carpinifolia* associated communities had a higher degree of overlap. One group of samples from *P. nigra* formed an isolated cluster, which was characterized by the predominance of Boletales (Figure S6) though there is no explanatory cause for this cluster from the experimental variables recorded.

**Fig. 6:**
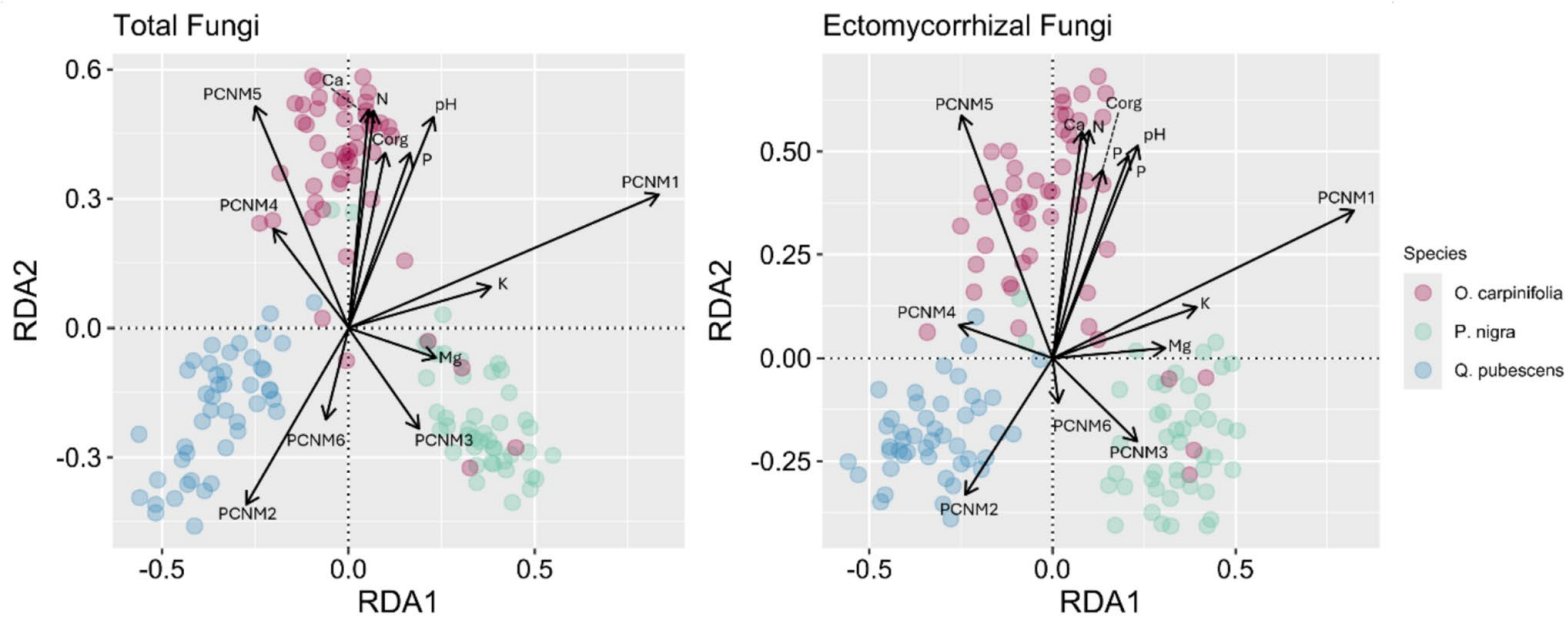
Redundancy analysis (RDA) ordination plots showing the relationship between community structure, soil parameters and geographical distance. Each dot represents a mesh bag community coloured by tree species as shown in the legend. Arrows represent seven soil parameters: pH, C_org_, total N, extractable P, Ca, Mg and K and geographical distance through PCNM vectors

When incorporating environmental variables (inclusive of soil parameters and geographical distance through PCNM vectors) using tbRDA analysis, they were found to be explanatory of 21.46% and 20.65% of the variation in total fungal community composition and EcM fungal community composition, respectively. Both tbRDA models were statistically significant (*p* = 0.001), and every considered variable that was included in the model was significant in its contribution to variation in total fungal community assembly, though Ca and PCNM6 were not found to significantly contribute to EcM community assembly (Table S8), though it is important to consider that Ca and N are highly co-linear. There is a complex inter-correlated relationship between the spatial location of plots, soil properties, and the identity of associated tree species (Fig. 6). Complementary Mantel testing performed comparing between-sample Euclidean distances with Bray–Curtis distances confirms that spatial layout of the plots contributes significantly to between-sample variance, though this correlation was found to be weak (total fungi r^2^ = 0.19, EcM fungi r^2^ = 0.24). Extractable Mg separated total fungal communities of *P. nigra* from those associated with the other two tree species. Matching the observed underlying variability in soil properties between *O. carpinifolia* and the two other tree species (soils associated with *O. carpinifolia* were higher in pH, C_org_, total N, extractable P and extractable Ca), soil pH, C_org_, N and Ca content separated total fungal communities of *O. carpinifolia* from *Q. pubescens and P. nigra*. Soil Mg and K content separated the total fungal communities of *P. nigra, O. carpinifolia* and *Q. pubescens*. Extractable soil Mg and K separated EcM fungal communities associated with *P. nigra* from those associated with the other two tree species. Soil C_org_, P, N and extractable Ca separated *O. carpinifolia* associated EcM communities from those of *Q. pubescens and P. nigra*. For soil parameters separated by plot location, see Figure S4 and Table S2.

**Fig. 7:**
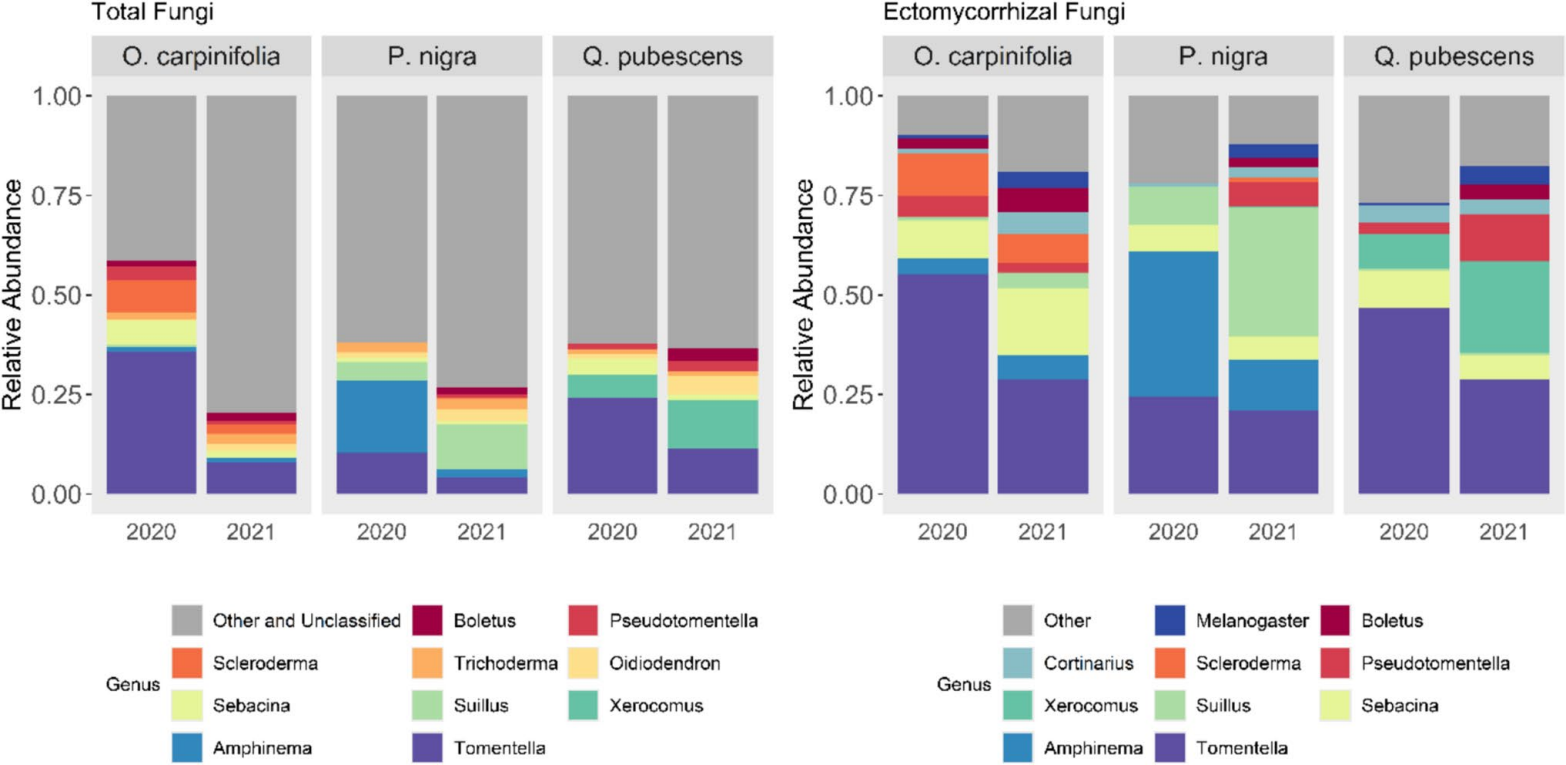
Taxa charts displaying the mean relative abundances of the ten most abundant genera for total fungi and EcM fungi observed colonizing mesh bags arranged by tree species and sampling campaign (i.e. year)

Of the top 10 most abundant genera across the total fungal dataset, all but *Trichoderma* and *Oidiodendron* were EcM (Fig. 7). The most abundant genera in *O. carpinifolia* and *Q. pubescens* were *Tomentella* and *Sebacina,* in *O. carpinifolia* they were joined by *Scleroderma* and by *Xerocomus* in *Q. pubescens*. In *P. nigra* the most abundant in 2020 was *Amphinema*, followed by *Tomentella* and *Suillus*, while in 2021 *Suillus* was highly abundant, followed by *Tomentella* and *Amphinema*.

### Shared and unique OTUs and genera associated with mesh bags across tree species

**Fig. 8:**
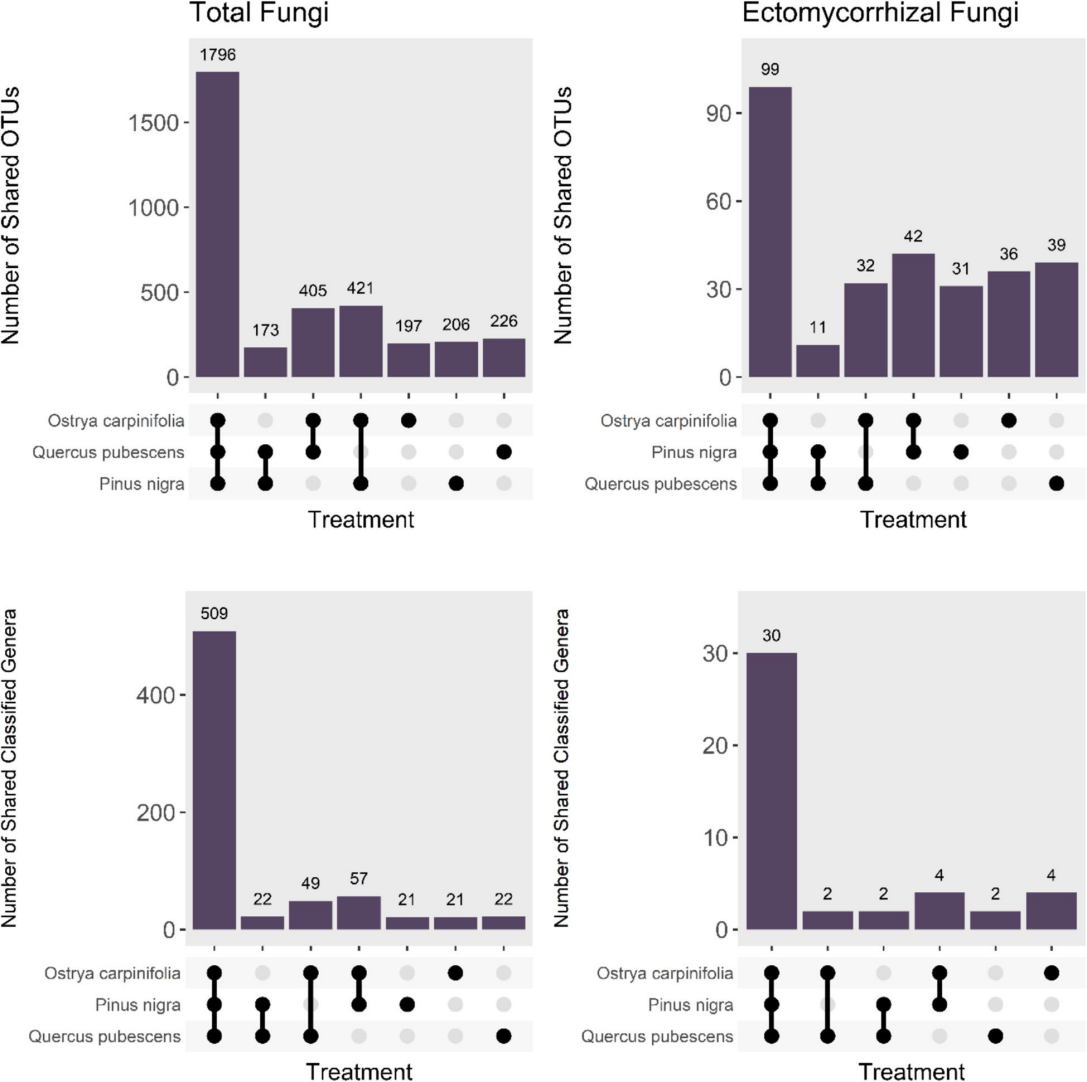
Shared and unique OTUs and genera for total fungi and EcM subset in mycelial communities of mesh bags

Among total fungi, 52.45% OTUs were shared by all three tree species, the percentage of shared OTUs that were associated with two tree species ranged between 5.05–12.30%, while 5.75–6.60% of OTUs were detected in only one tree species. Among the EcM fungal subset, 34.14% of OTUs were shared by all three tree species, 3.79–14.48% of OTUs were found with two species, while 10.69–13.45% of OTUs were specific for one tree species. The *Q. pubescens*-*P. nigra* pair exhibited lower numbers of shared OTUs compared to other two species pairs, both for total fungi and EcM fungi (Fig. 8). Across the 701 classified genera of total fungi and 44 classified genera of EcM fungi, 72.61% and 68.18% of genera were shared between all three tree species.

Indicator species analysis was performed considering only the total fungal community, as it would also reveal indicator genera within the EcM subset. In total, 40 indicator genera (Table S9) were identified across all tree species. EcM genera indicative of *O. carpinifolia* were *Scleroderma* (1 OTU, LD) and *Hebeloma* (1 OTU, SD). The *Tomentella* genus was associated with both *Q. pubescens* (9 OTUs) and *O. carpinifolia* (3 OTUs, SD). *Q. pubescens* was additionally associated with a mix of EcM fungi exhibiting varied exploration types including *Byssocorticium* (1 OTU, SD), *Xerocomus* (2 OTUs, LD)*, Hygrophorus* (1 OUT, SD) and *Amanita* (1 OTU MD). *P. nigra* was associated mostly with EcM taxa associated with MD and LD exploration including *Amphinema* (3 OTUs, MD), *Rhizopogon* (1 OTU, LD), *Suillus* (1 OTU, LD), *Polyozellus* (1 OTU, MD), and *Tricholoma* (2 OTUs, MD). The remaining associated genera exhibiting a broad range of ecologies were individually associated with *P. nigra* (two plant pathogen genera, six saprotrophic genera), *O. carpinifolia* (one plant pathogen genus, five saprotrophic genera, two genera of unknown ecology) and *Q. pubescens* (two plant pathogen genera, seven saprotrophic genera, one genus of unknown ecology) and with multiple combinations of tree species (Table S9).

## Discussion

In this study we investigated the variability in mycelium production, diversity and community composition of fungi from mesh bags beneath *Ostrya carpinifolia*, *Quercus pubescens* and *Pinus nigra* growing in Sub-Mediterranean Karst environment, in two consecutive years, where mesh bag incubation start and end differed in timing (spring vs. early summer). Additionally, EcM subset was characterized by the exploration types.

Our results indicate species-specific temporal trends in mycelium production. While *O. carpinifolia* showed a decline of mycelium quantity in bags retrieved in early summer 2021, both *P. nigra* and *Q. pubescens* responded positively, with significantly higher production levels in bags retrieved in early summer 2021. Although the cumulative precipitation was comparable between the two years, the distribution of precipitation was different, with the first year of mesh bag exposure experiencing relatively drier beginning of the year compared to the second year (see Figure S3). This aligns with the existing evidence indicating that the production of (EMM) mycelium is positively correlated to soil moisture (e.g. Söderström 1979; Castaño et al. 2017, Hagenbo et al. 2021), emphasizing the importance of precipitation patterns in regulating mycelial growth dynamics in forest ecosystems. In addition, specific adaptations of tree species to soil moisture limitations (Hagenbo et al. 2018) and seasonality of maximum carbon flow belowground (Štursova et al. 2020) may also govern the mycelium production and should be considered as possible reasons for the differences that we observed.

Although mesh bags were designed to selectively include only EcM fungi by intentional removal of organic C sources (Wallander et al. 2001), other fungal guilds were also detected. According to Hagenbo et al. (2018), the distribution between the functional groups in mesh bags incubated for approximately one year tends to reflect that of the surrounding soil. During this period, some mortality of EMM of EcM fungi could have occurred as the estimated mycelium longevity in Mediterranean forests ranges between 37–55 days (Hagenbo et al. 2021), which is substantially shorter than the mesh bag exposure time in our study. In addition, a process of adsorption of natural organic matter on sand quartz particles (Jada et al. 2006) through percolation and in the form of hyphal exudates might have occurred which could serve as a food for decomposer fungi. Other inputs of fungal material could be by spores shedding from sporocarps (Koide et al. 2005) into topsoil. Moreover, wild boar activity, which frequently disturbs the topsoil at the site, likely facilitated the mixing of fungal material from the surface into deeper soil layers, affecting the observed fungal diversity in the mesh bags. Despite these shortcomings, mesh bags filter taxa that can form EMM and thus represent an advantage compared to bulk soil analyses where mycelium-forming fungi cannot be separated from dormant fungi.

In 2021, the proportion of EcM fungi in mesh bags declined compared to 2020. However, this difference was significant only for *O. carpinifolia.* The difference in recovery timing of mesh bags between the two sampling campaigns (April for the first campaign and July for the second campaign) likely influenced these results, as EcM mycelium production is known to exhibit seasonal variability. A seasonality of mycelium production was reported for boreal and temperate forests (Wallander et al. 2001, Štursova et al. 2020). In Mediterranean, two peaks of EcM mycelium production were detected, first in October–November and the second at the end of February, while for summer months no data were provided (Hagenbo et al. 2021). In the second campaign the peak of EcM mycelium biomass might have been missed, potentially allowing ingrowth of saprotrophic mycelium at available niche. This shift is consistent with the higher relative abundance of saprotrophs in the mesh bags during second campaign. This pattern is further reflected in the OTU richness, where EcM fungal richness was generally lower in bags retrieved in early summer 2021 compared to bags retrieved in spring 2020. Conversely, total fungal OTU richness increased in bags retrieved in early summer 2021, particularly for *O. carpinifolia* and *P. nigra*, driven primarily by the proliferation of saprotrophic fungi. The question whether EcM fungi and saprotrophic fungi have different seasonal dynamics was already raised by Ekblad et al. (2013), but at that time the ecology of the many fungal taxa was unknown. Sequential colonisation might be a result of competitive or antagonistic interactions that occur between EcM fungi and saprotrophs, where EcM fungi might exert negative effects on decomposition (Leake et al. 2002; Cairney 2005).

Contrary to our expectations, *P. nigra* exhibited the lowest observed EcM OTU richness despite having thick roots that may struggle to access tiny soil niches, thus increasing its dependence on EcM fungi. The only EcM conifer in the area is a non-native *P. nigra*, introduced in the 19th century. Since no native EcM conifers grow in the vicinity to serve as a direct inoculum source for conifer-specific EcM fungi, *P. nigra* might had limited access to more diverse fungal community. The introduction of tree species into new areas often leads to substantial reduction of EcM species richness (Vlk et al. 2020). Introduced Pinaceae almost exclusively form symbiosis with co-introduced EcM in regions where no native Pinaceae are present (Vlk et al. 2020). However, in some cases, Pinaceae are able to form EcM associations with native EcM fungi present at the site of introduction, such as *Sebacina* and Thelephoraceae (Vlk et al. 2020). Although *P. nigra* was introduced, all three species at our site shared around 70% of EcM and total fungal OTUs in mesh bags. Interestingly, *P. nigra* and *Q. pubescens* shared fewer EcM OTUs than *P. nigra* and *O. carpinifolia*, a pattern also observed for the total fungi. This could partly be explained by the lower proximity of some sampling sites for *P. nigra* and *O. carpinifolia* compared to *P. nigra* and *Q. pubescens*. However, since *Q. pubescens* and *O. carpinifolia* sites were also distant from each other yet shared higher number of OTUs compared to *P. nigra* and *Q. pubescens* pair, spatial distance alone does not fully account for these findings. Therefore, we can only partly confirm our hypothesis that *Q. pubescens* and *O. carpinifolia* would share a higher number of OTUs in mycelial communities compared to the pairs of both broadleaved species with conifer.

Despite high proportion of shared OTUs, all investigated tree species had relatively distinct mycelial communities of total fungi. However, differences were less pronounced for EcM mycelia, especially between *P. nigra* and *O. carpinifolia*, which clustered together. This pattern may be attributed to the spatial proximity of two *P. nigra* sampling locations to one *O. carpinifolia* location suggesting that the spatial component plays a significant role in shaping EcM mycelial communities, whereas it appears to have less influence on the composition of other fungal mycelia. As a result, trees in closer proximity have a higher likelihood of sharing EcM mycelial communities, which in turn facilitates the formation of mycorrhizal networks**.** Our previous observations from ECM root tip and EMM of *Q. pubescens* in this area revealed substantial dissimilarities in the community composition of EcM fungi among the plots (Mrak et al. 2021, 2025). Apparently, the fragmented nature of forest in the area prevents mycelium to overcome the distances between the fragments, while the input from other inoculation sources appears to be largely stochastic. This indicates an island effect (Peay et al. 2010), where priority is an important determinant of EcM community structure.

In EMM communities of all tree hosts, *Tomentella* was one of the most dominant components. *Tomentella* is one of the most common EcM genera in forests worldwide (Jakucs and Eros-Honti 2008) and was also one of the most abundant and species rich taxa in the investigated area on *Q. pubescens* roots (Mrak et al. 2021, 2025). Similarly, in our previous study in this area, *Tomentella* was also the most dominant component of mesh-bag mycelia of *Q. pubescens*, along with *Sebacina* and *Pseudotomentella* (Mrak et al. 2021). Although different *Tomentella* species may classify under contact, SD or MD exploration types (Agerer 2001), SD morphotypes are predominantly observed on tree roots of *Q. pubescens* in this area (Mrak et al. 2025). However, this apparent limitation in exploration strategy does not prevent *Tomentella* from being a dominant component of mycelia in mesh bags. Notably, many EcM genera that are typically considered to produce low amount of EMM based on their exploration type can still extensively colonise mesh bags (Jörgensen et al. 2023). The relative abundance of mycelia from different genera in mesh bags also reflects differences in mycelial turnover rates (Jörgensen et al. 2023). Longer exposure periods may favour taxa with slower turnover rates, leading to increased detection over time. For example, rhizomorphs tend to be more long-lived than solitary emanating hyphae (Ekblad et al. 2013) which may result in higher abundances of rhizomorph-forming taxa after longer mesh bag exposure times. Furthermore, caution should be used when interpreting taxon abundance using PCR amplification of fungal rDNA due to high level of variation in the ITS copy numbers among fungal taxa (Baldrian et al. 2013).

Surprisingly, despite their high frequency and abundance on *Q. pubescens* root tips of in this area—as confirmed by our temporal studies in 2016–2018 and 2021–2022 (Mrak et al. 2021, 2025)—*Cenococcum* and Pyronemataceae (*Genea*, *Humaria*) were either absent or poorly represented in mesh bags, despite the fact that they belong to SD exploration types, similarly as *Tomentella*. Nevertheless, we detected many saprotrophic Dothideomycetes (Ascomycota), where *Cenococcum* is the only known EcM representative, as well as *Cenococcum* was reported from mesh bags by Hagenbo et al. (2018). This suggests that ecological factors may have contributed to the lower representation of certain *Ascomycota* taxa in the mesh bags.

Among EcM indicators genera, *Amphinema*, *Rhizopogon* and *Suillus* were almost exclusively found in mesh bags under *P. nigra*. These genera are highly specialized EcM fungi that predominantly associate with Pinaceae (Dahlberg and Finlay 1999; Erland and Taylor 1999; Molina et al. 1999; Bruns et al. 2002; Tedersoo et al. 2024). However, smaller amounts of *Suillus and Amphinema* mycelia were also detected in mesh bags under *O. carpinifolia*, which could be explained by the proximity of some *P. nigra* trees to our sampling plots for *O. carpinifolia*. Since *Suillus* is specific for Pinaceae (Dahlberg and Finlay 1999; Tedersoo et al. 2024), its presence below *O. carpinifolia* trees suggests potential competition between the mycelial communities of *O. carpinifolia* and *P. nigra* for the uptake of water and nutrients. *Suillus, Amphinema and Rhizopogon* all produce abundant mycelia and are classified as MD and LD exploration types (Agerer 2001), enabling them to efficiently absorb and transport water and nutrients from the parts of soil several decimetres away from the tree roots (Agerer 2001), thus indicating higher spatial spread of EMM of *P. nigra*. These fungi could be very beneficial for the absorption of water and nutrients in *P. nigra* due to its thick roots. However, studies have shown that despite forming abundant mycelia, *Suillus* and *Rhizopogon* exhibit relatively low levels of actual colonization of tree roots (Bruns et al. 2002). Therefore, we should be careful about the conclusions on the benefits that these fungi may bring as they may be more resource consuming for the tree host due to their specialized nature (Bruns et al. 2002). Indicator EcM fungi of *O. carpinifolia* and *Q. pubescens* in our study are all known to associate with a wide array of different hosts, including *Pinus* (Brand 1989; Kottke et al. 1998; Tedersoo et al. 2024). *Xerocomus*, an indicator of *Q. pubescens* in our study, is classified as LD exploration type EcM fungus (Agerer 2001), but despite its ability to form large genets with approximately 100 m in diameter (Fiore-Donno and Martin 2001), its mycelia did not occur under *P. nigra* and *O. carpinifolia*. This could be due to the fragmented nature of forest in this area, which is interspersed with meadows and pastures. Spreading over meadows does not bring any benefit to EcM fungus as no suitable hosts are found there.

Only two non-ectomycorrhizal genera were detected among ten most abundant genera of total fungi, *Oidiodendron and Trichoderma*, both forming filamentous mycelia (Põlme et al. 2021). *Oidiodendron* was assigned to the soil saprotroph functional group but may also occur as an ericoid mycorrhizal fungus (Martino et al. 2018). *Trichoderma* was co-assigned to the mycoparasite and foliar endophyte functional groups (Põlme et al. 2021). In addition, it was also reported to grow on roots and infect outer root cells, where it induces plant defence responses (Harman et al. 2004). This is resulting in host resistance to plant pests, tolerance to abiotic stresses, increased plant growth and photosynthetic capability, and contributes towards the solubilization of nutrients (Harman et al. 2004; del Carmen et al. 2021). Some endophytes may continue their life cycles as saprotrophs, thereby contributing significantly to the first stages of litter decomposition (Saikkonen et al. 2015), which explains why *Trichoderma* was often considered as a soil saprotroph (del Carmen et al. 2021).

There were also quite a few saprotrophs among indicator genera, mainly belonging to wood saprotrophs, which could be associated with the specific wood qualities of all three species, particularly the presence of resin in *P. nigra*. *Sarea*, an indicator genus of *P. nigra*, is a resinicolous fungus, found only in Pinaceae (Mitchell et al. 2021). Similarly, *Lophium* species are mainly reported from conifers and may also grow on resin (Czachura and Janik 2024). Some species of *Gymnopus,* another indicator genus of *Pinus*, are fruiting on pine needles (Petersen and Hughes 2016). All three genera form filamentous mycelia (Põlme et al. 2021).

Soil properties and spatial distance determined around 20% variation in the communities of the total fungal mycelium and EcM mycelium developed in the mesh bags despite the relatively small spatial scale of the study, where generally no big variations in soil characteristics were encountered. The topsoils under *Q. pubescens* and *P. nigra* were relatively similar, but *O. carpinifolia* showed higher values of pH, C_org_, N and extractable Ca, which were discriminatory in separating its community from the other two species. As *O. carpinifolia* grows on remnants of drywalls, constructed of limestone, higher extractable Ca levels, which are normally associated with higher pH values (Monfort-Salvador et al. 2015), are reasonable. Experiments with liming have revealed significant changes in EcM communities and identified liming as a major determinant of EcM community structure, even stronger than the host species (Rineau and Garbaye 2009). On the other hand, during our fieldwork we noticed large accumulations of leaf litter and humus under *O. carpinifolia*, which is consistent with higher C_org_ and N levels observed for soil associated with this tree species. Moreover, exchangeable Ca was identified as a key factor controlling soil C and N following agricultural abandonment in Chinese karst by improving soil organic matter stability (Li et al. 2017). Both, C_org_ and N are very important in structuring the soil fungal communities (Chen et al. 2021; Qi et al. 2021). Based on these observations, for *O. carpinifolia* we cannot separate the effects of tree identity from the effects of soil properties on the community composition of total fungi and the EcM subset.

Host tree species is an important determinant of the relative abundance of exploration types (Rosinger et al. 2018; Fernandez et al. 2023) and our observations confirm this co-dependence. The high abundance of SD exploration types in EMM of *Q. pubescens* was consistent with our previous observations on exploration types of root-tip associated ECM communities of *Q. pubescens* in this area, determined by the combination of morphotyping and molecular methods (Mrak et al. 2021). However, for the other two tree species no prior data on the abundance of exploration types or EcM community composition were available from this area. For *O. carpinifolia* generally, the knowledge on EcM fungal communities is limited to *Tuber* cultivation (Benucci et al. 2011; Baciarelli Falini et al. 2012; Moser et al. 2017), while the study by Fernandez et al. (2023), which investigated the exploration types in some *Pinus* species was performed in temperate climate but included the effects of reduced soil moisture and increased temperature. In their study, *Pinus strobus* was associated with higher abundance of MD-LD distance exploration types in EMM, mainly *Suillus*, which is consisted with our findings. The higher abundance of exploration types with higher spatial spread in *P. nigra* could be related to the relative thickness of *P. nigra* fine roots compared to both broad-leaved species in our study (Chen et al. 2018). Thicker roots have limited access to tiny soil pores to forage for water and nutrients, making fungal hyphae and rhizomorphs essential for resource acquisition (Chen et al. 2016). Long distance exploration types are associated with higher C costs for their formation (Rosinger et al. 2018) but they tend to be longer-living and may accumulate substantial biomass over time (Jörgensen et al. 2023).

Interannual variability in the share of exploration types was also observed, which could be explained by the variability in meteorological conditions (Rosinger et al. 2018; Mrak et al. 2021; Fernandez et al. 2023). Previously studies on the root-tip community of ECM fungi of *Q. pubescens* in this ecosystem identified a negative correlation of SD exploration type with precipitation, positive correlation of LD exploration type with precipitation, and negative correlations of MD exploration type with soil temperature and air humidity (Mrak et al. 2021). Spring 2021 had more precipitation compared to spring 2020, which could be associated with higher share of LD and lower share of SD exploration types in mycelial communities of all three host species in 2021. This suggests that annual climatic variability plays a role in shaping the composition and functional strategies of EcM mycelial communities.

## Conclusions

Our study highlights the complex interactions between host tree species, and environmental factors in shaping mycelial communities in a Sub-Mediterranean Karst environment. Despite the high proportion of shared fungal OTUs among tree species, mycelial communities remained relatively distinct. Distinct soil properties associated with *O. carpinifolia* prevented the separation of host effect from the effect of soil properties, while this was possible for *P. nigra* and *Q. pubescens*. Tree host identity and interannual fluctuations possibly related to seasonal effects and/or precipitation patterns particularly affected the mycelium production and balance between short- and long-distance exploration types. *P. nigra* exhibited unexpectedly low EcM OTU richness, likely due to its non-native status and lack of compatible inoculum sources. The presence of saprotrophic fungi in EcM mesh bags suggests secondary colonization, driven by decomposition dynamics and potential competitive interactions between EcM fungi and saprotrophs. Overall, our findings emphasize the importance of host specificity, soil properties, spatial proximity, and climatic variability in structuring mycelial communities in fragmented forests. Further research is needed to clarify the mechanisms underlying competitive interactions between fungal guilds and their impact on nutrient cycling and tree health.

## Supplementary Information

Below is the link to the electronic supplementary material.

Supplementary file1 (PDF 775 KB)

## Data Availability

Data and metadata supporting the findings of this study are available via DiRROS repository (10.20315/Data.0004). Raw sequence data were deposited in SRA (BioProject PRJNA1243991).
