# Supplementary material for: Mycelial communities associated with Ostrya carpinifolia, Quercus pubescens and Pinus nigra in a patchy Sub-Mediterranean Karst woodland
Source: Mycorrhiza. 2025 Jul 25;35(4):46. doi: 10.1007/s00572-025-01220-9 (PMC12296906; doi:10.1007/s00572-025-01220-9)
Supplement: Supplementary file 1 — Supplementary file1 (PDF 775 KB) [file 572_2025_1220_MOESM1_ESM.pdf]

Supplementary material: Mycelial communities associated with *Ostrya carpinifolia*, *Quercus pubescens* and *Pinus nigra* in a patchy Sub-Mediterranean Karst woodland

Tanja Mrak[1*], Philip Alan Brailey-Crane[2], Nataša Šibanc[1], Tijana Martinović[1], Jožica Gričar[1], Hojka Kraigher[1]

[1]Slovenian Forestry Institute, Department of Forest Genetics and Physiology, Večna pot 2, 1000 Ljubljana, Slovenia
[2]University of Georgia, Department of Genetics, C424 Life Sciences Building, Athens, GA 30602, USA

*corresponding author

Supplementary Figures

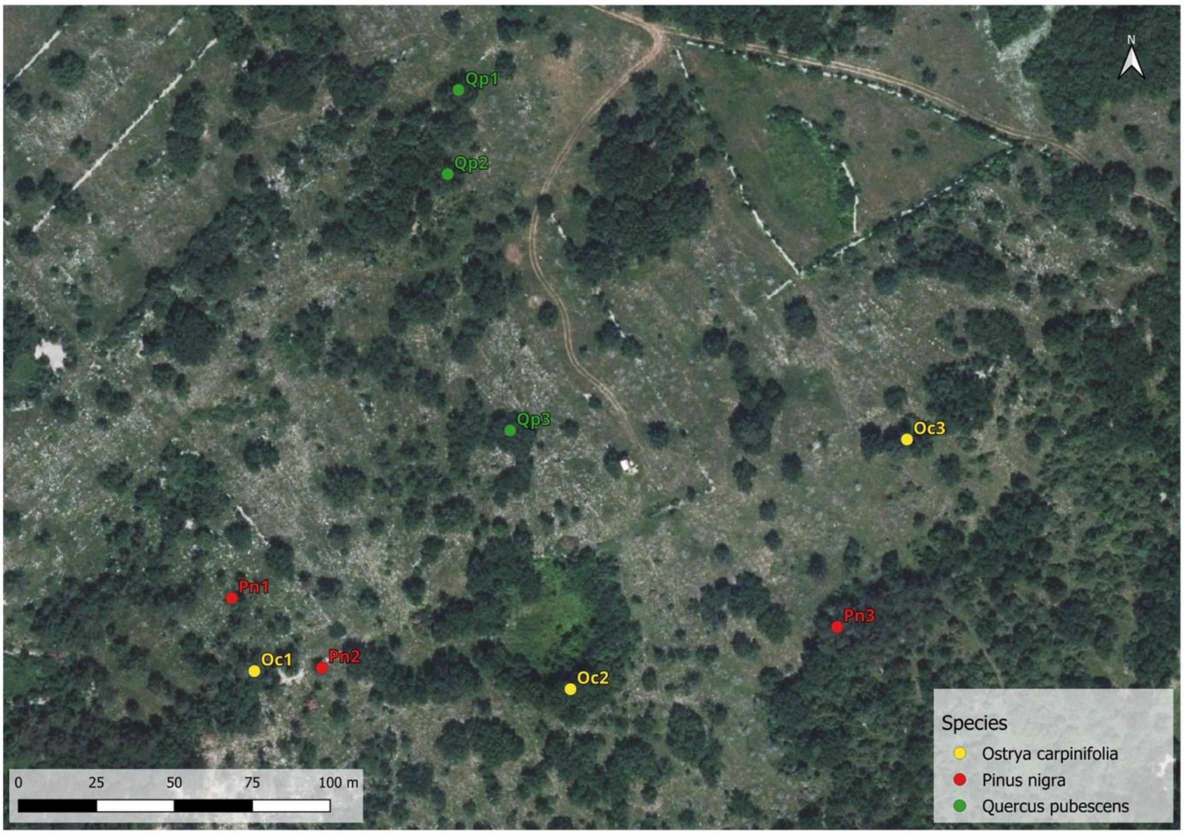

Figure S1: Locations of the experimental plots where the mesh bag experiment was conducted in Podgorski kras, Slovenia. Studied tree species are shown in different colors

a) b)

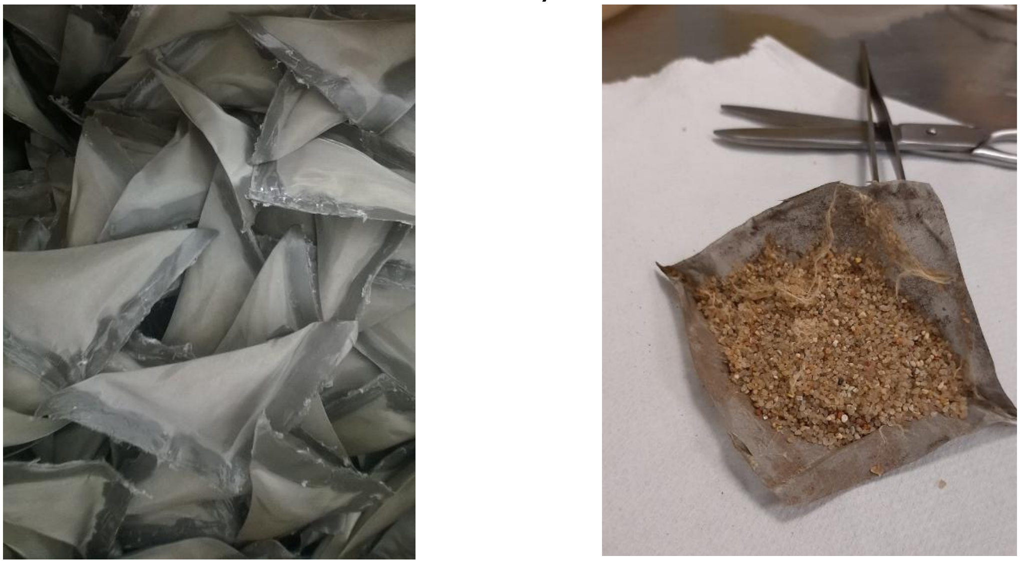

Figure S2: a) In-growth mesh bags b) In-growth mesh bags after field incubation, estimation of mycelium quantity

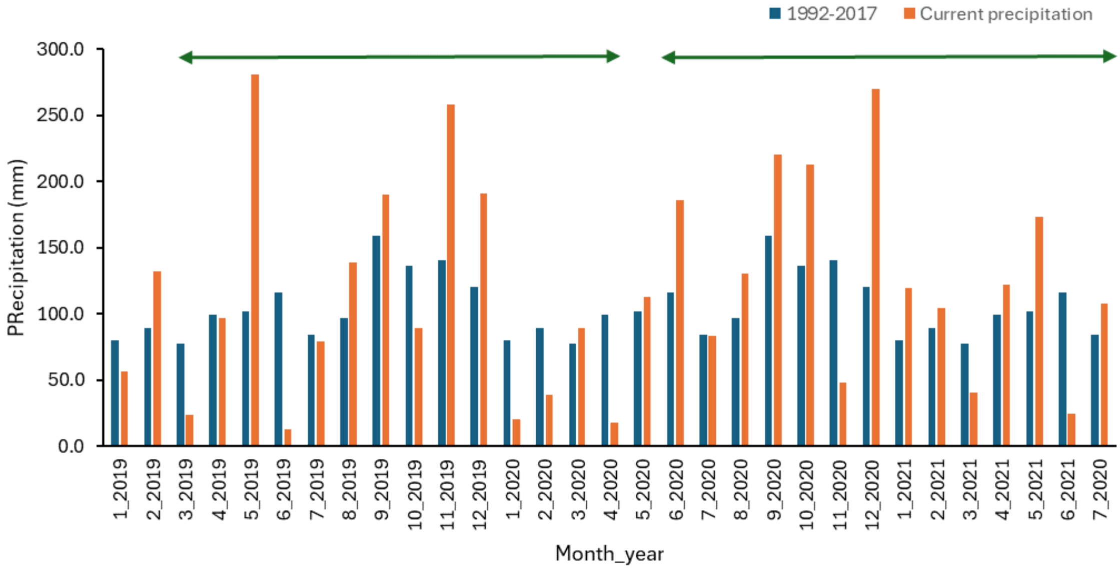

Figure S3: Precipitation for Kozina weather station (Slovenia, ARSO) in the timeframe of the mesh bag experiment in comparison with 25-year average monthly precipitation (1992-2017). Arrows mark the periods from the mesh bag installation to their recovery from the soil

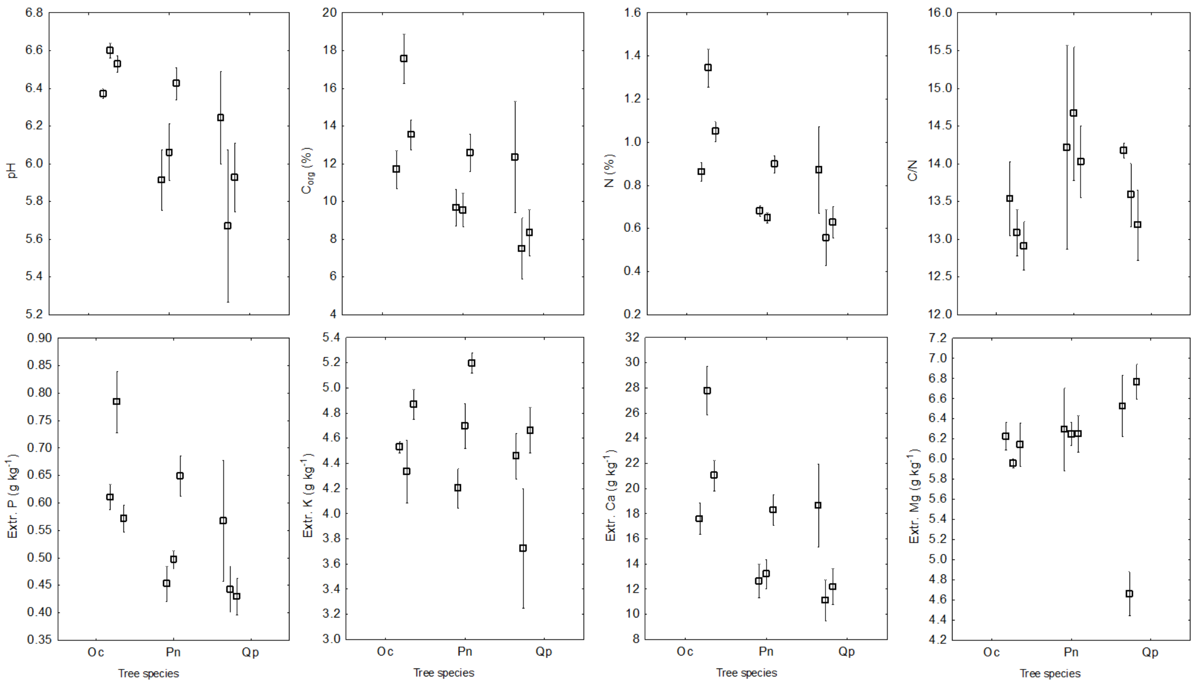

Figure S4: Variability of soil parameters among tree species *Ostrya carpinifolia* (Oc), *Pinus nigra* (Pn) and *Quercus pubescens* (Qp), and among the plots (N = 3 plots per species). For each plot three pooled samples were analyzed (shown as mean ± SE)

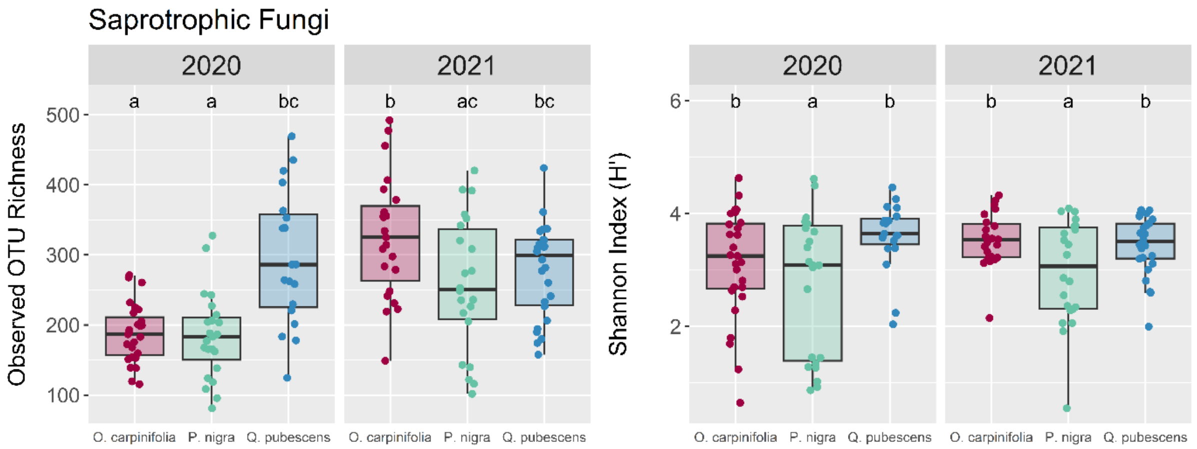

Figure S5: Boxplots displaying the distribution of observed OTU richness and Shannon diversity index (H') for the saprotroph subset. Boxes cover the 25th-75th percentile of each group's distribution, with the median represented as a thick bar within the boxes. Extending lines from the box denote the 1.5 interquartile range of the 25th and 75th percentile. Significantly different values ($p < 0.05$) are marked with different letters. Replications per subset: *O. carpinifolia* 2020 n= 26, *O. carpinifolia* 2021 n=23, *P. nigra* 2020 n=23, *P. nigra* 2021 n=24, *Q. pubescens* 2020 n= 19, *Q. pubescens* 2021 n=26

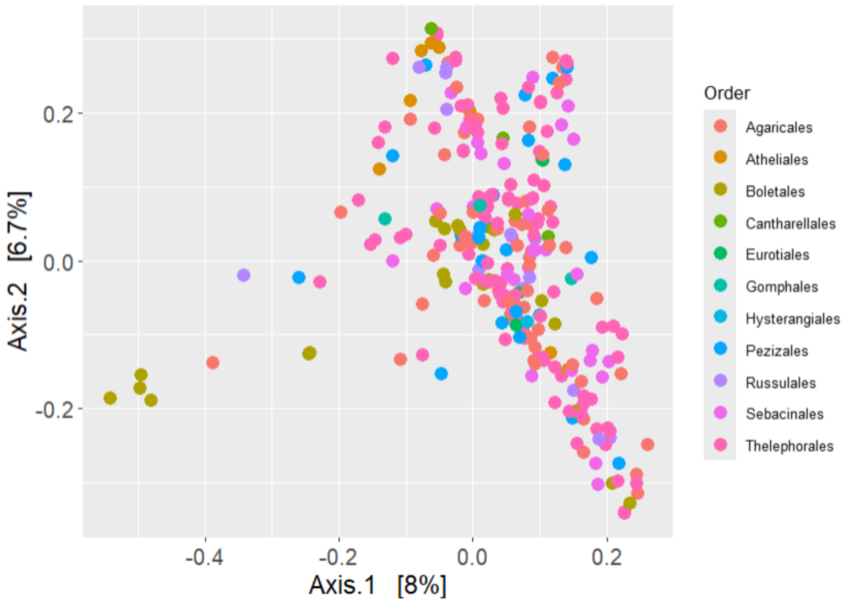

Figure S6: Principal co-ordinate analysis (PCoA) of EcM fungal communities associated with Figure 5 in the main text. Instead of site scores, the OTU scores are displayed, colored by order-level taxonomy

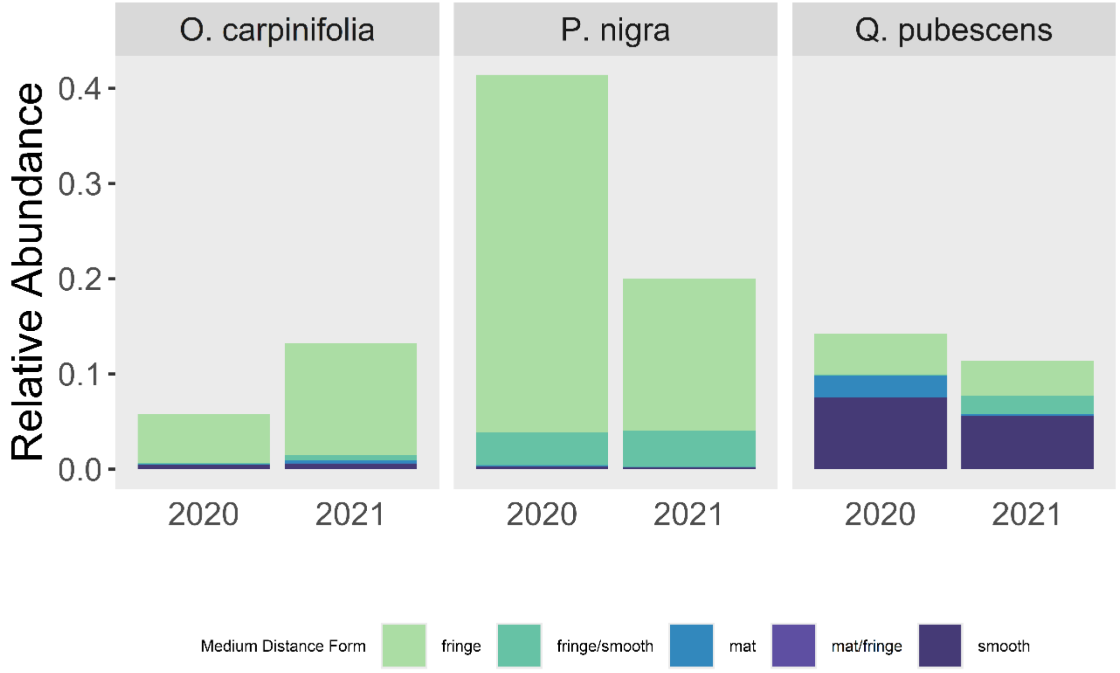

Figure S7: Mean relative abundances of medium distance sub-types of ectomycorrhizal fungi detected in mycelium communities of mesh bags incubated beneath *Ostrya carpinifolia*, *Pinus nigra* and *Quercus pubescens* in 2020 and 2021

Supplementary Tables

Table S1: Coordinates of the plots for the mesh bag experiment at Podgorski kras, Slovenia

Tree species	Plot ID	latitude	longitude
Quercus pubescens	Qp1	45.5425505	13.9168970
Quercus pubescens	Qp2	45.5423074	13.9168570
Quercus pubescens	Qp3	45.5415704	13.9171279
Pinus nigra	Pn1	45.5410762	13.9159938
Pinus nigra	Pn2	45.5408784	13.9163682
Pinus nigra	Pn3	45.5410167	13.9184819
Ostrya carpinifolia	Oc1	45.5408666	13.9160907
Ostrya carpinifolia	Oc2	45.5408270	13.9173900
Ostrya carpinifolia	Oc3	45.5415600	13.9187581

Table S2: Soil edaphic properties presented as mean ± SE values analyzed for soils associated with each tree species. For each plot three pooled samples were analyzed. Different lowercase letters indicate significantly different values at p<0.05

	***Ostrya carpinifolia* (N = 9)**	***Pinus nigra* (N = 9)**	***Quercus pubescens* (N = 9)**
pH	6.501 ± 0.038 **a**	6.133 ± 0.102 **ab**	5.947 ± 0.168 **b**
Total C	14.287 ± 1.016 **a**	10.626 ± 0.693 **b**	9.421 ± 1.281 **b**
$CaCO_3$	0.196 ± 0.028	0.159 ± 0.033	0.214 ± 0.099
Mineral C	0.023 ± 0.004	0.020 ± 0.003	0.026 ± 0.012
Organic C	14.266 ± 1.013	10.604 ± 0.690	9.402 ± 1.278
Total N	1.0850 ± 0.0770 **a**	0.7428 ±0.0419 **b**	0.6857 ± 0.0866 **b**
Extractable P	0.6554 ± 0.0377 **a**	0.5329 ± 0.0331 **b**	0.4796 ± 0.0417 **b**
Extractable K	4.5778 ± 0.1126	4.6986 ± 0.1610	4.2803 ± 0.2110
Extractable Ca	22.1393 ± 1.6750 **a**	14.7118 ± 1.0926 **b**	13.9840 ± 1.6344 **b**
Extractable Mg	6.1101 ± 0.08426	6.2637 ± 0.1348	5.9856 ± 0.3538
texture	Clay	Clay	Clay

Table S3 Results of the linear models testing the effect of tree species on soil properties. Values at p < 0.05 are highlighted in **bold**

Variable	df	F-value	P-value
pH	24,2	5.76	**9.08E-03****
Total C	24,2	6.11	**7.16E-03****
$CaCO_3$	22,2	0.26	0.77
Mineral C	22,2	0.2	0.82
Organic C	24,2	6.14	**0.01***
Total N	24,2	9.22	**1.07E-03****
Extractable P	24,2	5.72	**9.29E-03****
Extractable K	24,2	1.67	0.21
Extractable Ca	24,2	9.16	**1.11E-03****
Extractable Mg	24,2	0.39	0.68

Table S4: Linear model results assessing the variability of functional guild relative abundances between tree species and sampling year. Values at $p < 0.05$ are highlighted in **bold**. Degrees of freedom: Species / Species x Year = 2,134; Year = 1,134

Guild	Factor	F-value	P-value
Ectomycorrhiza	Species	1.71	0.19
	Year	34.83	**2.79×10^{-8}** ***
	Species x Year	9.66	**1.21×10^{-4}** ***
Plant Pathogen	Species	2.80	0.06
	Year	13.38	**3.64×10^{-4}** ***
	Species x Year	9.45	**1.44×10^{-4}** ***
Saprotrophs	Species	3.71	**0.03** *
	Year	46.77	**2.57×10^{-10}** ***
	Species x Year	0.84	0.43

Table S5. Linear model results assessing the variability of ectomycorrhizal exploration type relative abundances between tree species and sampling year. Values at $p < 0.05$ are highlighted in **bold**

Exploration Type	Df	Factor	F-value	P-value
Long-Distance	134,2	Species	1.11	0.33
	134,1	Year	11.27	**1.00×10^{-03}** **
	134,2	Species x Year	0.67	0.51
Medium Distance *(All Forms)*	134,2	Species	10.99	**3.82×10^{-05}** ***
	134,1	Year	0.27	0.6
	134,2	Species x Year	3.16	**0.45×10^{-1}** *
Short Distance	134,2	Species	14.34	**2.26×10^{-06}**
	134,1	Year	8.65	**3.85×10^{-03}**
	134,2	Species x Year	0.8	0.45
Contact	134,2	Species	0.34	0.71
	134,1	Year	0.09	0.76
	134,2	Species x Year	3.36	**0.04** *
Medium Distance *(Fringe)*	134,2	Species	17.13	**2.38E-07** ***
	134,1	Year	2.01	0.16
	134,2	Species x Year	6.24	**2.56E-03** **
Medium Distance *(Smooth)*	134,2	Species	5.9	**3.50E-03** **
	134,1	Year	6.57	**0.01** *
	134,2	Species x Year	2.47	0.09
Medium Distance *(Mat)*	134,2	Species	1.04	0.36
	134,1	Year	0.83	0.36
	134,2	Species x Year	1.46	0.24
Medium Distance *(Fringe/Smooth)*	133,2	Species	3.39	**0.04** *
	133,1	Year	0.08	0.78
	133,2	Species x Year	0.08	0.92
Medium Distance *(Mat/Fringe)*	129,2	Species	2.14	0.12
	129,1	Year	0.1	0.75
	129,2	Species x Year	0.71	0.49

Table S6: Variability of alpha diversity metrics for total fungi, ectomycorrhizal subset and saprotroph subset considering the effect of tree species and year. Values at $p<0.05$ are highlighted in **bold**. Degrees of freedom: Species / Species x Year = 2,134; Year = 1,134

Alpha diversity parameter	Factor	Total fungi	EcM fungi	Saprotroph fungi
Observed richness	Species	F=9.64 **p=1.23 x 10^{-4***}**	F=16.55 **p=3.96 x 10^{-4***}**	F=8.604 **p=3.05 x 10^{-4***}**
	Year	F=6.24 **p=0.01***	F=21.78 **p=7.33 x 10^{-6***}**	F=9.05 **p=3.14 x 10^{-3**}**
	Species x Year	F=10.89 **p=4.13 x 10^{-5***}**	F=0.56 p=0.58	F=8.79 **p=2.59 x 10^{-4***}**
Shannon diversity index	Species	F=0.92 p=0.40	F=1.54 p=0.22	F=6.95 **p=1.34 x 10^{-3**}**
	Year	F=35.36 **p=2.25 x 10^{-8***}**	F=0.31 p=0.58	F=1.42 p=0.24
	Species x Year	F=4.79 **p=9.79 x 10^{-3***}**	F=0.32 p=0.72	F=1.42 p=0.24

Table S7: Intraspecific variation in community assembly for each tree species. Values at $p<0.05$ are highlighted in bold

		Total fungi		Ectomycorrhizal fungi	
Tree species	Factor	F	p	F	p
O. carpinifolia	Location	2.3454	**0.001*** **	2.66136	**0.001*** **
	Year	4.0648	**0.001*** **	2.1909	**0.001*** **
	Location*year	1.3233	**0.050* **	1.2177	0.163
P. nigra	Location	2.4888	**0.001*** **	1.8676	**0.011* **
	Year	2.5370	**0.001*** **	2.6109	**0.007** **
	Location*year	1.1403	0.198	0.7173	0.880
Q. pubescens	Location	2.3293	**0.001*** **	1.9686	**0.001*** **
	Year	1.7794	**0.007** **	1.1257	0.306
	Location*year	0.9513	0.601	0.6387	0.987

Table S8: Significance of variables incorporated into RDA for total fungi and EcM subset. Variance (%) represents the proportion of total inertia explained by each contributing variable scaled from 0-100

	Total fungi				Ectomycorrhizal fungi			
	Inertia	Variance (%)	F	P-value	Inertia	Variance (%)	F	P-value
pH	0.02	1.95	3.12	**0.001***	0.02	2.06	3.3	**0.001***
Corg	0.01	1.07	1.7	**0.002**	0.01	1.16	1.85	**0.001***
N	0.01	1.75	2.79	**0.001***	0.01	2.01	3.2	**0.001***
P	0.01	1.36	2.17	**0.001***	0.01	1.36	2.17	**0.001***
K	0.01	1.42	2.26	**0.001***	0.01	1.34	2.14	**0.001***
Ca	0.01	0.86	1.37	**0.021**	0.01	0.77	1.23	0.093
Mg	0.01	1.25	2	**0.001***	0.01	1.02	1.63	**0.011**
PCNM1	0.03	3.83	6.11	**0.001***	0.03	4.01	6.4	**0.001***
PCNM2	0.01	1.56	2.49	**0.001***	0.01	1.24	1.98	**0.001***
PCNM3	0.01	1.14	1.82	**0.001***	0.01	1.42	2.27	**0.001***
PCNM4	0.01	1.51	2.4	**0.001***	0.01	1.22	1.94	**0.001***
PCNM5	0.02	2.35	3.75	**0.001***	0.02	2.62	4.18	**0.001***
PCNM6	0.01	1	1.59	**0.008**	0.01	0.83	1.33	0.059
Constrained	0.18	21.46			0.15	20.65		
Unconstrained	0.66	78.96			0.57	78.94		
Total	0.84	100			0.73	100		

Table S9a. Indicator Genera for mesh bags associated with one or a combination of tree species. Mean relative abundances ± SE are given for each tree species. P-values have been FDR corrected for multiple testing. Ecological guild was obtained from Põlme et al. (2021) unless marked with an asterisk. For ectomycorrhizal guild exploration type is given in brackets (SD – short distance, MD – medium distance, LD – long distance)

Genus	O. carpinofolia	P. nigra	Q. pubescens	Indicator	Indicator Statistic	P-value	Ecological Guild
Scleroderma	0.060 ± 0.020	8.170e-04 ± 8.120e-04	3.020e-06 ± 2.080e-06	O. carpinofolia	0.26	0.019	Ectomycorrhizal(LD)
Hebeloma	0.010 ± 0.005	3.400e-04 ± 2.140e-04	9.900e-04 ± 4.540e-04	O. carpinofolia	0.26	0.006	Ectomycorrhizal(SD)
Tomentella	0.230 ± 0.030	0.070 ± 0.020	0.170 ± 0.030	O. carpinofolia + Q. pubescens	0.3	0.006	Ectomycorrhizal(SD)
Rhizopogon	9.770e-05 ± 6.120e-05	0.020 ± 0.010	1.710e-05 ± 7.380e-06	P. nigra	0.19	0.003	Ectomycorrhizal(LD)
Amphinema	0.010 ± 0.006	0.100 ± 0.030	8.540e-06 ± 5.610e-06	P. nigra	0.4	0.003	Ectomycorrhizal(MD (fringe))
Polyozellus	4.840e-06 ± 2.750e-06	0.010 ± 0.006	0	P. nigra	0.2	0.003	Ectomycorrhizal(MD)
Suillus	0.004 ± 0.004	0.080 ± 0.030	1.500e-04 ± 1.920e-05	P. nigra	0.34	0.003	Ectomycorrhizal(LD)
Tricholoma	3.030e-04 ± 2.260e-04	0.020 ± 0.010	0.002 ± 0.002	P. nigra	0.2	0.033	Ectomycorrhizal (MD smooth)
Byssocorticium	0	0	0.020 ± 0.010	Q. pubescens	0.24	0.003	Ectomycorrhizal(SD)
Xerocomus	3.280e-06 ± 2.580e-06	2.150e-05 ± 1.610e-05	0.090 ± 0.020	Q. pubescens	0.42	0.003	Ectomycorrhizal(LD)
Hygrophorus	5.590e-06 ± 3.340e-06	6.610e-06 ± 6.610e-06	0.005 ± 0.005	Q. pubescens	0.13	0.003	Ectomycorrhizal(SD)
Amanita	0.002 ± 0.001	1.200e-04 ± 8.330e-05	0.030 ± 0.010	Q. pubescens	0.32	0.003	Ectomycorrhizal(MD (smooth))
Dothiorella	0.004 ± 8.950e-04	0.001 ± 6.120e-04	2.610e-04 ± 5.770e-05	O. carpinofolia	0.32	0.003	Plant Pathogen
Diplodia	0.001 ± 7.380e-04	0.008 ± 0.002	1.060e-04 ± 3.490e-05	P. nigra	0.37	0.003	Plant Pathogen
Venturia	0.002 ± 0.002	0.030 ± 0.005	5.650e-05 ± 5.080e-05	P. nigra	0.51	0.003	Plant Pathogen
Phialophora	4.100e-05 ± 4.040e-05	3.350e-06 ± 2.400e-06	0.004 ± 0.004	Q. pubescens	0.13	0.014	Plant Pathogen
Scleromitrula	9.770e-05 ± 2.780e-05	1.130e-05 ± 6.930e-06	0.004 ± 0.003	Q. pubescens	0.14	0.003	Plant Pathogen
Pyrenochaeta	0.006 ± 9.940e-04	0.002 ± 4.160e-04	0.003 ± 7.160e-04	O. carpinofolia	0.3	0.003	Saprotroph (wood)
Montagnula	0.003 ± 7.360e-04	2.270e-04 ± 1.550e-04	1.490e-05 ± 7.910e-06	O. carpinofolia	0.41	0.003	Saprotroph (wood)
Angustimassarina	0.004 ± 0.001	5.460e-04 ± 3.230e-04	5.570e-04 ± 1.720e-04	O. carpinofolia	0.27	0.009	Saprotroph (wood + mycopar.)
Pseudocamarosporium	0.010 ± 0.002	0.002 ± 7.700e-04	0.002 ± 3.480e-04	O. carpinofolia	0.41	0.003	Saprotroph (wood)
Kochiomyces	0.020 ± 0.004	0.003 ± 0.001	0.004 ± 0.001	O. carpinofolia	0.31	0.003	Saprotroph (pollen)
Coniophora	3.200e-05 ± 1.030e-05	0.040 ± 0.020	1.580e-05 ± 9.830e-06	P. nigra	0.29	0.003	Saprotroph (wood)
Sarea	0.001 ± 6.860e-04	0.007 ± 0.002	1.080e-04 ± 1.010e-04	P. nigra	0.38	0.003	Saprotroph (resin*)
Mytilinidion	2.060e-04 ± 1.130e-04	0.005 ± 0.002	2.760e-06 ± 1.660e-06	P. nigra	0.27	0.003	Saprotroph (wood)
Cryptosporiopsis	0.001 ± 3.200e-04	0.010 ± 0.008	0.001 ± 2.240e-04	P. nigra	0.18	0.003	Saprotroph (unspecified)
Lophium	0.001 ± 7.770e-04	0.005 ± 0.001	4.250e-05 ± 2.070e-05	P. nigra	0.32	0.003	Saprotroph (litter)
Gymnopus	0.000e+00 ± 0.000e+00	0.010 ± 0.010	1.110e-05 ± 8.840e-06	P. nigra	0.13	0.048	Saprotroph (litter)
Oidiodendron	0.009 ± 0.002	0.020 ± 0.006	0.030 ± 0.007	P. nigra + Q. pubescens	0.22	0.048	Saprotroph (soil)
Preussia	0.002 ± 0.001	0.001 ± 5.220e-04	0.020 ± 0.007	Q. pubescens	0.28	0.003	Saprotroph (soil & dung, endophyte**)
Neopyrenochaeta	6.220e-04 ± 3.010e-04	0.001 ± 3.750e-04	0.003 ± 7.480e-04	Q. pubescens	0.33	0.003	Saprotroph (unspecified)
Humicola	0.002 ± 2.680e-04	0.001 ± 3.640e-04	0.005 ± 6.330e-04	Q. pubescens	0.45	0.003	Saprotroph (wood)
Virgaria	5.540e-04 ± 1.810e-04	5.770e-05 ± 1.480e-05	0.006 ± 0.001	Q. pubescens	0.54	0.003	Saprotroph (wood & litter)
Saitozyma	0.001 ± 2.410e-04	1.970e-04 ± 5.660e-05	0.005 ± 8.410e-04	Q. pubescens	0.52	0.003	Saprotroph (soil)
Talaromyces	0.001 ± 3.350e-04	0.002 ± 8.430e-04	0.006 ± 0.001	Q. pubescens	0.34	0.003	Saprotroph (unspecified)
Paraphaeosphaeria	3.070e-04 ± 9.800e-05	0.002 ± 6.080e-04	0.020 ± 0.007	Q. pubescens	0.25	0.003	Saprotroph (wood)
Phlegmacium	0.020 ± 0.010	1.350e-05 ± 5.140e-06	1.370e-04 ± 7.190e-05	O. carpinofolia	0.17	0.026	Unknown
Keithomyces	0.003 ± 3.880e-04	9.880e-04 ± 2.150e-04	0.001 ± 1.860e-04	O. carpinofolia	0.37	0.003	Unknown
Penicillago	0.005 ± 0.002	0.007 ± 0.002	7.980e-04 ± 3.210e-04	O. carpinofolia + P. nigra	0.22	0.043	Unknown

Genus	O. carpinofolia	P. nigra	Q. pubescens	Indicator	Indicator Statistic	P-value	Ecological Guild
Thelephora	8.670e-05 ± 5.620e-05	8.810e-07 ± 8.810e-07	0.020 ± 0.007	Q. pubescens	0.28	0.003	Unknown

* Mitchell, J.K., Garrido-Benavent, I., Quijada, L. et al. Sareomycetes: more diverse than meets the eye. IMA Fungus 12, 6 (2021). https://doi.org/10.1186/s43008-021-00056-0

** Arenal, F., Platas, G. & Peláez, F. 2007. A new endophytic species of Preussia (Sporormiaceae) inferred from morphological observations and molecular phylogenetic analysis. Fungal Diversity 25: 1–17.

Table S9b. Indicator OTUs for mesh bags associated with one or a combination of tree species. Unique OTU_ID refers to the number given to each OTU in deposited metadata (10.20315/Data.0004). P-values have been fdr corrected for multiple testing. OTU genus and ecological guild membership (Põlme et al. 2021) have been included to facilitate interpretation. For ectomycorrhizal guild exploration type is given in brackets (SD – short distance, MD – medium distance, LD – long distance)

OTU	Unique OTU_ID	Indicator	Indicator Statistic	P-value	Genus	Ecological Guild
Sebacina cystidiata OTU 1	OTU24	O. carpinofolia	0.22	0.02	Sebacina	Ectomycorrhizal (SD)
Unclassified Sebacina OTU 12	OTU25	O. carpinofolia	0.21	0.01	Sebacina	Ectomycorrhizal (SD)
Scleroderma areolatum OTU 1	OTU18	O. carpinofolia	0.26	0.02	Scleroderma	Ectomycorrhizal (LD)
Hebeloma erebium OTU 1	OTU79	O. carpinofolia	0.23	0.04	Hebeloma	Ectomycorrhizal (SD)
Unclassified Tomentella OTU 41	OTU28	O. carpinofolia	0.24	0.01	Tomentella	Ectomycorrhizal (SD)
Tomentella umbrinospora OTU 3	OTU3	O. carpinofolia	0.33	0.003	Tomentella	Ectomycorrhizal (SD)
Unclassified Tomentella OTU 82	OTU8	O. carpinofolia	0.26	0.01	Tomentella	Ectomycorrhizal (SD)
Unclassified Amphinema OTU 1	OTU1	P. nigra	0.29	0.003	Amphinema	Ectomycorrhizal (MD fringe)
Unclassified Amphinema OTU 6	OTU6	P. nigra	0.3	0.003	Amphinema	Ectomycorrhizal (MD fringe)
Rhizopogon mohelnensis OTU 1	OTU26	P. nigra	0.2	0.003	Rhizopogon	Ectomycorrhizal (LD)
Suillus granulatus OTU 1	OTU9	P. nigra	0.34	0.003	Suillus	Ectomycorrhizal (LD)
Unclassified Amphinema OTU 4	OTU21	P. nigra	0.22	0.03	Amphinema	Ectomycorrhizal (MD fringe)
Polyozellus rhizopunctatus OTU 1	OTU77	P. nigra	0.14	0.03	Polyozellus	Ectomycorrhizal (MD)
Unclassified Tomentella OTU 88	OTU98	P. nigra	0.22	0.003	Tomentella	Ectomycorrhizal (SD)
Tricholoma terreum OTU 2	OTU304	P. nigra	0.22	0.003	Tricholoma	Ectomycorrhizal (MD smooth)
Tricholoma batschii OTU 1	OTU43	P. nigra	0.17	0.05	Tricholoma	Ectomycorrhizal (MD smooth)
Unclassified Sebacina OTU 3	OTU127	Q. pubescens	0.16	0.01	Sebacina	Ectomycorrhizal (SD)
Melanogaster broomeanus OTU 1	OTU785	Q. pubescens	0.23	0.01	Melanogaster	Ectomycorrhizal (LD)
Xerocomus subtomentosus OTU 1	OTU16	Q. pubescens	0.4	0.003	Xerocomus	Ectomycorrhizal (LD)
Xerocomus subtomentosus OTU 2	OTU815	Q. pubescens	0.24	0.003	Xerocomus	Ectomycorrhizal (LD)
Cortinarius epipurrus OTU 1	OTU316	Q. pubescens	0.13	0.003	Cortinarius	Ectomycorrhizal (MD fringe)
Unclassified Amanita OTU 1	OTU56	Q. pubescens	0.32	0.003	Amanita	Ectomycorrhizal (MD smooth)
Byssocorticium atrovirens OTU 1	OTU107	Q. pubescens	0.24	0.003	Byssocorticium	Ectomycorrhizal (SD)
Hygrophorus persoonii OTU 1	OTU206	Q. pubescens	0.13	0.003	Hygrophorus	Ectomycorrhizal (SD)
Tomentella punicea OTU 1	OTU1056	Q. pubescens	0.15	0.04	Tomentella	Ectomycorrhizal (SD)
Unclassified Tomentella OTU 13	OTU131	Q. pubescens	0.23	0.03	Tomentella	Ectomycorrhizal (SD)
Unclassified Tomentella OTU 45	OTU31	Q. pubescens	0.16	0.003	Tomentella	Ectomycorrhizal (SD)
Unclassified Tomentella OTU 52	OTU45	Q. pubescens	0.16	0.02	Tomentella	Ectomycorrhizal (SD)
Unclassified Tomentella OTU 54	OTU46	Q. pubescens	0.18	0.01	Tomentella	Ectomycorrhizal (SD)
Unclassified Tomentella OTU 57	OTU48	Q. pubescens	0.22	0.003	Tomentella	Ectomycorrhizal (SD)

OTU	Unique OTU_ID	Indicator	Indicator Statistic	P-value	Genus	Ecological Guild
Unclassified Tomentella OTU 59	OTU51	Q. pubescens	0.19	0.003	Tomentella	Ectomycorrhizal (SD)
Unclassified Tomentella OTU 60	OTU52	Q. pubescens	0.24	0.003	Tomentella	Ectomycorrhizal (SD)
Unclassified Tomentella OTU 77	OTU75	Q. pubescens	0.19	0.003	Tomentella	Ectomycorrhizal (SD)
Lecanicillium psalliotae OTU 1	OTU151	P. nigra	0.4	0.003	Lecanicillium	Other
Metapochonia rubescens OTU 1	OTU123	Q. pubescens	0.25	0.01	Metapochonia	Other
Unclassified Dothiorella OTU 1	OTU173	O. carpinofolia	0.32	0.003	Dothiorella	Plant Pathogen
Unclassified Diplodia OTU 1	OTU110	P. nigra	0.37	0.003	Diplodia	Plant Pathogen
Unclassified Venturia OTU 4	OTU30	P. nigra	0.51	0.003	Venturia	Plant Pathogen
Unclassified Ceratobasidium OTU 7	OTU93	Q. pubescens	0.2	0.04	Ceratobasidium	Plant Pathogen
Phialophora cyclaminis OTU 1	OTU126	Q. pubescens	0.13	0.003	Phialophora	Plant Pathogen
Unclassified Scleromitrula OTU 1	OTU136	Q. pubescens	0.14	0.003	Scleromitrula	Plant Pathogen
Angustimassarina acerina OTU 1	OTU295	O. carpinofolia	0.27	0.01	Angustimassarina	Saprotroph
Unclassified Kochiomyces OTU 1	OTU211	O. carpinofolia	0.23	0.04	Kochiomyces	Saprotroph
Kochiomyces dichotomus OTU 1	OTU62	O. carpinofolia	0.32	0.003	Kochiomyces	Saprotroph
Unclassified Montagnula OTU 3	OTU242	O. carpinofolia	0.41	0.003	Montagnula	Saprotroph
Unclassified Pseudocamarosporium OTU 1	OTU83	O. carpinofolia	0.41	0.003	Pseudocamarosporium	Saprotroph
Unclassified Pyrenochaeta OTU 2	OTU171	O. carpinofolia	0.23	0.02	Pyrenochaeta	Saprotroph
Unclassified Cladophialophora OTU 25	OTU257	O. carpinofolia + Q. pubescens	0.35	0.003	Cladophialophora	Saprotroph
Coniophora fusispora OTU 1	OTU122	P. nigra	0.2	0.003	Coniophora	Saprotroph
Coniophora arida OTU 2	OTU17	P. nigra	0.2	0.003	Coniophora	Saprotroph
Unclassified Cryptosporiopsis OTU 2	OTU163	P. nigra	0.15	0.003	Cryptosporiopsis	Saprotroph
Lophium arboricola OTU 1	OTU176	P. nigra	0.3	0.003	Lophium	Saprotroph
Unclassified Odontia OTU 1	OTU409	P. nigra	0.19	0.003	Odontia	Saprotroph
Sarea resinae OTU 1	OTU140	P. nigra	0.39	0.003	Sarea	Saprotroph
Myrmecridium schulzeri OTU 1	OTU134	P. nigra + Q. pubescens	0.22	0.04	Myrmecridium	Saprotroph
Unclassified Cladophialophora OTU 34	OTU441	Q. pubescens	0.35	0.003	Cladophialophora	Saprotroph
Unclassified Exophiala OTU 13	OTU87	Q. pubescens	0.3	0.003	Exophiala	Saprotroph
Unclassified Humicola OTU 1	OTU177	Q. pubescens	0.47	0.003	Humicola	Saprotroph
Unclassified Neopyrenochaeta OTU 1	OTU153	Q. pubescens	0.33	0.003	Neopyrenochaeta	Saprotroph
Unclassified Paraphaeosphaeria OTU 1	OTU200	Q. pubescens	0.29	0.003	Paraphaeosphaeria	Saprotroph
Unclassified Paraphaeosphaeria OTU 3	OTU53	Q. pubescens	0.19	0.01	Paraphaeosphaeria	Saprotroph
Preussia flanaganii OTU 1	OTU114	Q. pubescens	0.29	0.01	Preussia	Saprotroph
Unclassified Preussia OTU 4	OTU222	Q. pubescens	0.32	0.003	Preussia	Saprotroph
Unclassified Preussia OTU 6	OTU372	Q. pubescens	0.18	0.003	Preussia	Saprotroph
Saitozyma podzolica OTU 1	OTU277	Q. pubescens	0.53	0.003	Saitozyma	Saprotroph
Talaromyces minioluteus OTU 1	OTU366	Q. pubescens	0.4	0.003	Talaromyces	Saprotroph
Virgaria nigra OTU 1	OTU186	Q. pubescens	0.48	0.003	Virgaria	Saprotroph
Phlegmacium pseudocephalixum OTU 1	OTU88	O. carpinofolia	0.17	0.03	Phlegmacium	Unknown
Unclassified Chalara OTU 2	OTU160	O. carpinofolia	0.2	0.01	Chalara	Unknown

OTU	Unique OTU_ID	Indicator	Indicator Statistic	P-value	Genus	Ecological Guild
Keithomyces carneus OTU 1	OTU139	O. carpinofolia	0.37	0.003	Keithomyces	Unknown
Unclassified Hypocreales OTU 11	OTU106	O. carpinofolia	0.29	0.01	Unclassified	Unknown
Unclassified Gloniaceae OTU 1	OTU19	O. carpinofolia	0.28	0.003	Unclassified	Unknown
Unclassified Dothideomycetes OTU 40	OTU220	O. carpinofolia	0.23	0.03	Unclassified	Unknown
Unclassified GS17 OTU 2	OTU235	O. carpinofolia	0.23	0.02	Unclassified	Unknown
Unclassified Cephalothecaceae OTU 3	OTU326	O. carpinofolia	0.15	0.01	Unclassified	Unknown
Unclassified Pleosporales OTU 97	OTU80	O. carpinofolia	0.36	0.003	Unclassified	Unknown
Penicillago nodositata OTU 1	OTU65	O. carpinofolia + P. nigra	0.22	0.03	Penicillago	Unknown
Unclassified Chalara OTU 7	OTU221	O. carpinofolia + Q. pubescens	0.22	0.03	Chalara	Unknown
Unclassified Lasionectriopsis OTU 1	OTU133	O. carpinofolia + Q. pubescens	0.4	0.003	Lasionectriopsis	Unknown
Linnemannia amoeboidea OTU 1	OTU330	P. nigra	0.22	0.01	Linnemannia	Unknown
Unclassified Pleosporales OTU 26	OTU124	P. nigra	0.3	0.01	Unclassified	Unknown
Unclassified Sordariaceae OTU 1	OTU129	P. nigra	0.29	0.01	Unclassified	Unknown
Unclassified Eurotiales OTU 1	OTU13	P. nigra	0.29	0.003	Unclassified	Unknown
Unclassified Atheliaceae OTU 2	OTU36	P. nigra	0.22	0.003	Unclassified	Unknown
Unclassified Agaricomycetes OTU 11	OTU5	P. nigra	0.34	0.003	Unclassified	Unknown
Unclassified Dothideomycetes OTU 63	OTU72	P. nigra	0.28	0.003	Unclassified	Unknown
Unclassified Thyridariaceae OTU 1	OTU81	P. nigra	0.37	0.003	Unclassified	Unknown
Unclassified Didymellaceae OTU 5	OTU96	P. nigra	0.14	0.03	Unclassified	Unknown
Thelephora atra OTU 1	OTU64	Q. pubescens	0.28	0.003	Thelephora	Unknown
Oidiodendron chlamydosporicum OTU 1	OTU97	Q. pubescens	0.41	0.003	Oidiodendron	Unknown
Unclassified Hyaloscyphaceae OTU 1	OTU104	Q. pubescens	0.29	0.003	Unclassified	Unknown
Unclassified Delitschiaceae OTU 2	OTU141	Q. pubescens	0.33	0.003	Unclassified	Unknown
Unclassified Sporormiaceae OTU 5	OTU142	Q. pubescens	0.19	0.003	Unclassified	Unknown
Unclassified Ascomycota OTU 35	OTU15	Q. pubescens	0.25	0.003	Unclassified	Unknown
Unclassified Ascomycota OTU 45	OTU167	Q. pubescens	0.31	0.003	Unclassified	Unknown
Unclassified Chaetomiaceae OTU 2	OTU185	Q. pubescens	0.28	0.01	Unclassified	Unknown
Unclassified Sordariales OTU 35	OTU67	Q. pubescens	0.16	0.003	Unclassified	Unknown
